# Periscope of Authenticated Hospital Reports, *English and Foreign*, with Commentaries

**Published:** 1826-10-01

**Authors:** 


### Periscope

OF

### AUTHENTICATED HOSPITAL REPORTS,

### English and Foreign,

### WITH COMMENTARIES.

On the utility and importance of authentic histories of cases occurring in public institutions, we have uniformly insisted, and we are now very happy to see that several of our most distinguished metropolitan physicians and surgeons have liberally, and we think wisely, adopted the resolution, not only of recording the more important cases occurring in their respective departments, in a book open to the inspection of all the students, but of permitting these documents to be diffused through the profession at large, by means of the press.* This has been acted on for some years in the Parisian hospitals, and the beneficial effects are acknowledged all over the Continent. Whatever scepticism may attach to statements made by individuals in private practice—(a scepticism, however, which we think is now carried by many people to an unjust degree,) the records of hospitals, preserved in the above manner, both at home and abroad, are perfectly free from suspicion of infidelity. Any attempt at adulterating, or even colouring cases of this kind, would argue little else than insanity in the superior medical officer; while, from those who are appointed to copy or abstract the histories, all temptation of misrepresentation is not only taken away, but a positive danger would be incurred by such attempt—the reporter being known, and, of course, responsible for errors or inaccuracies—much more for perversions of facts. From what we have ourselves seen, the same remarks apply, with strict justice, to the records and recorders of foreign hospitals, where matters of this kind are arranged and laid open to inspection, with still more care than in this country.

In our Periscope of Hospital Reports we hope to give as much

* It has been most untruly asserted that we are inconsistent in our conduct, having, it is alleged, at one time, condemned the publication of hospital reports. We deny it. We condemned, and do still condemn, *garbled and erroneous reports*, as injurious and unjust; but we have ever recommended, in the most strenuous terms, the publication of *authenticated*, *correct*, and *fair reports* of cases occurring in public hospitals. If this be inconsistency, we hope never to act otherwise.

satisfaction to all parties as in our analyses of other species of medical literature—namely, by conveying the more important or useful facts to our readers, notwithstanding the abbreviations which must necessarily take place, but which habit enables us to make, without material detriment to, much less disfiguration of, the original documents.

In our commentaries, we shall always endeavour to be guided, not only by strict regard for truth and justice, but by a religious horror of that prostitution, or rather profanation of the pen of criticism which, by ribaldry, perversion, or abuse, would crush every attempt to open the flood-gates of knowledge, excepting for our own personal advantage, and roll back to its source the tide of information, rather than permit it to flow through any other channel than our own. But, while we cautiously avoid all approach to such degrading and unprincipled conduct, we shall hold ourselves free to comment on such points of doctrine or practice as appear to invite remark; and that, in the spirit of liberality and delicacy which befits a science, having the mitigation of human suffering for its object and end.

With these few preliminary observations we proceed to our task, and hope that our actions will not be found to fall short of our professions.

---

### 1. M. LISFRANC ON WHITE SWELLING.*

ALTHOUGH we cannot expect much novelty on the subject of white swellings, after the investigations in this country by a Russel, a Crowther, and more especially a Brodie; yet it will not be uninteresting to know the opinions, the researches, and the modes of treatment of M. Lisfranc, who has talent to observe, and a wide field for observation. We shall therefore present our readers with a brief account of the present memoir, omitting the detail of cases, amounting to twenty-two in number.

Our author defines white swelling to be an *engorgement*, generally slow and dense, of the soft parts forming or surrounding the joints—with or without alteration of structure in the bones and cartilages. M. Lisfranc's observations lead him to believe that the disease may commence in any one of the tissues of the joint—but that it generally begins in the ligaments—the cartilages—or the bones, whence it spreads more or less rapidly to those structures which are most contiguous.

M. L. divides the disease into two classes—idiopathic and symptomatic. He thinks the minute divisions and distinctions into which Mr. Brodie has gone, are useless, if not injurious.

*Idiopathic* white swelling is that which succeeds external violence, in an individual otherwise healthy. This class, M. L. avers, is equally as common as the other, whatever may be said to the contrary. This species always begins in the soft parts. The *symptomatic* white swelling is that which grows up under the influence of a morbid constitutional affection, as scrofula, gout, and rheumatism. These are the *only* diseases to which he could trace white swelling. Authors, however, have endea-

* Memoire sur les Tumeurs Blanches des Articulations; recueilli à l'Hôpital de la Pitié, dans les Salles de M. Lisfranc, &c.—*Archives*, Mai, 1826.

voured to connect it with small-pox, measles, syphilis, the suppression or retrocession of other affections, &c. This species may commence either in the soft parts of the joint, or in the articulating extremities of the bones. In the beginning, both species are attended by inflammation, whether actute or chronic—but arrived at a certain epoch, that is, when the tissues are reduced into one homogeneous, or lardaceous mass, there is no symptom of inflammation to be seen, except the *swelling* which is far from being a proof of that process. The symptoms are the same, both in the symptomatic and idiopathic species, when the disease commences in the soft parts; symptoms so well known that M. Lisfranc does not stop to describe them. He observes, however, that when the disease is produced by rheumatism or by external violence, the form of the inflammation is generally acute—though never so rigid in its march as common phlegmonous inflammation. The skin is rarely reddened. We come, therefore, to the pathology—or morbid anatomy of white swelling.

*Pathological Anatomy.* Six cases came under M. Lisfranc's observation, where the patients were cut off by other diseases, at a more or less early period of the white swelling. In all of these, the alterations were of the *same kind*—and only differed in respect to *plus* and *minus.* But to proceed from the surface to the interior. The skin was sound and free from inflammation in most of these cases—so was the subjacent cellular membrane, except that it contained a small quantity of serous infiltration, and was itself rather thicker than in a sound joint. Beneath this was found a yellow stratum more consistent than the cellular membrane, and interspersed with a number of vessels carrying red blood. In this stratum too, Mr. Lisfranc sometimes observed small tubercles. Still deeper, as the ligamentous structure was approached, this cellular bed or stratum became indurated, lardaceous, and resistant to the scalpel. The articular ligaments were distended with a liquid more or less consistent, and sometimes gellatinous. The capsular ligaments or bags were of a reddish colour—often much thickened, and containing a fluid of variable consistence. In two or three instances M. Lisfranc found the articulating ends of the bones diseased—being increased in volume, softened in substance, and turned of a yellow colour. The cartilages participated in this state.

The following was the morbid anatomy of amputated members—that is, of the advanced state of white swelling.

The skin was sometimes thicker, sometimes thinner than natural—cellular membrane indurated, lardaceous, and confounded with the ligaments and capsular bags—the neighbouring portions of muscle pale, extenuated, and the interstitial cellular substance more or less infiltrated with albuminous matters—the trunks of nerves in the vicinity appeared enlarged and harder than natural—the veins dilated and varicose—the arteries enlarged, and their tunics thickened—depôts of purulent matter more or less numerous—ulcerations of the cartilages and denudations of the heads of the bones—capsular ligaments thickened or ulcerated—the bones tumefied, carious, softened. In the most advanced stage the whole joint is often a confused mass, in which it is almost impossible to recognize a trace of natural structure, except the tendons, and some portions of muscle.

In respect to diagnosis we need say nothing, as the disease cannot be mistaken. The prognosis is grave—except where the white swelling is idiopathic, or owing to rheumatism, in which cases there are hopes of cure, unless the state of disorganization is very far advanced indeed. The scrofulous white swelling is, of course, the most dangerous.

*Treatment.* Whether the disease commences in the hard or in the soft parts, it matters not, as far as the treatment is concerned—it is the same in both cases. M. Lisfranc avers that the acute inflammation of white swelling is never removed at once—it always terminates in the chronic form of the disease, and it is through this process alone that a cure is to be expected.

In the first place, whether the white swelling be acute or chronic, it is necessary to examine into the state of the thoracic and abdominal viscera. If there be any organic disease, in either of these cavities, it will be dangerous to attempt a cure of the white swelling. M. Lisfranc has repeatedly seen the visceral disease, in such cases, increase proportionally as the local affection yielded. Under these circumstances, we are advised by our author to content ourselves with combating the more urgent symptoms only of the white swelling, while we direct our attention to the more important visceral malady.

When there is nothing in the constitution to forbid the cure of the local disease, the first thing indispensible is absolute rest of the part, without which, nothing can be done. Anchylosis is always a probable effect or consequence, and therefore the position of the limb should be such as may render that event as little detrimental to the patient as possible. In order to prevent anchylosis, however, our author advises the practitioner to gently bend and extend the member every two or three days, when this can be done without giving or increasing the pain. Whenever gentle motion produces this effect, we may suspect that the process of anchylosis has commenced, and then we are to endeavour to promote rather than retard it. When purulent depôts have formed, they should be opened and evacuated as speedily as possible.

*Acute White Swelling.* General and local depletion ought here to be employed in proportion to the acuteness of the inflammation, and the strength of the patient. In the course of the first eight or ten days, two general bleedings should be taken, and from 30 to 50 leeches applied every two or three days. Fomentations and cataplasms are recommended in this stage to be assiduously employed, well impregnated with opium, after each sanguineous depletion. The regimen should, of course, correspond with the other antiphlogistic measures.

In the majority of cases, these means will, in the course of ten days, reduce the acute inflammation, and bring it into a chronic state. But sometimes this will not be the case—and then the question arises, how long are we to go on with the energetic depletive process? After the above period, M. Lisfranc advises that some food be given, first, of the lightest kind, chiefly the farinaceæ—and then, gradually more and more nutritive, otherwise we may considerably injure the tone of the digestive organs. In respect to local bleedings, they must be continued as long as they appear to afford relief to the part, and not to enfeeble too much the constitution. Sometimes, though very rarely, these means fail to quell the acute inflammation, and we are forced to desist for a time, and allow the patient to rally a little. The topical applications, in the mean time, are to be continued, and a moderate diet allowed. In a fortnight or three weeks, we may resume the local bleeding. Should all these means fail in reducing the march of the acute inflammations, there will be little other chance of saving the patient's life but by amputation. Many practitioners recommend this operation to be delayed till the patient is arrived at an advanced degree of emaciation. M. Lisfranc is of a very different opinion. He removes the limb as soon as he finds the means above-mentioned fail, and the patient's general health begins to be sensibly deteriorated. We think he is quite right.

*Chronic White Swelling.* When the acute has terminated (as it usually does) in the chronic form of the disease, free sanguineous depletion is detrimental. When the disease, however, has *commenced* in the chronic form, as it sometimes does, then M. Lisfranc thinks it advisable to employ a few active local depletions—since in this, as in several other parts of the body, there is sometimes a *latent* acute inflammation going on, which does not manifest itself by the usual symptoms. A remarkable instance of this kind happened a few years ago, the anatomical preparation being presented to the Academy of Medicine by M. Lisfranc. A vigorous young man was received into La Pitié, for a white swelling of the knee joint. He complained of no pain, even when the joint was bended or extended—in short, he did not present a single symptom of inflammation, except the *swelling*. While in the hospital he got intoxicated one day, and, falling on his head, he was carried off in a few hours. On dissection of the joint there were unequivocal evidences of a violent acute inflammation. The articular capsule was of a dark red colour, thickened, and softened in texture. The cellular tissue was yellow, abundant, hardened, and lardaceous, intersected by numerous red vessels. In this case it is evident that had stimulating applications been used at the beginning, under the impression that no active inflammation existed, the disease would have been greatly exasperated, and probably the limb would have been lost. Nevertheless the cure of chronic white swelling cannot be effected without stimulants, or resolvents, although their *modus operandi*, where there is disorganization or complete change of structure, is an enigma which we cannot at present solve. The fact, however, is certain, whatever may be the explanation.

The first step of treatment, then, in chronic white swelling, is the application of a small number of leeches—from two to six, with the precaution not to let the bites bleed long. On this plan, their action is rather excitant than otherwise, as has been proved hundreds of times by M. Lisfranc, at La Pitié. After the application of the number above-mentioned, with the precaution respecting the bites, we generally find, the next day, that the volume of the swelling is augmented—its sensibility increased—and sometimes an erysipelas established around the bites. These symptoms need occasion no alarm. In forty-eight hours the erysipelas will have disappeared—the tumefaction and sensibility will have abated—and the original volume of the swelling will be found to have become somewhat diminished. At the end of four or five days the leeching (on the small scale above-mentioned) may be renewed, and will be followed by similar effects. The temporary increase of excitement, after the leechings, was always regarded by M. Lisfranc as advantageous, being invariably, when moderate in degree, succeeded by either diminution of size, or softening in the consistence of the joint affected. The leechings should not, of course, be renewed till the excitement produced by the preceding batch has subsided. Sometimes the excitement rises too high after these small leechings, and continues more than two or three days. In that case, we must subdue the excitement by the application of a considerable number of leeches—which will rarely fail to produce the effect desired. Speaking generally, the after-excitement will be in an inverse ration to the number of leeches applied:—consequently, if five leeches produce too much subsequent inflammation, eight or ten ought to be applied the next time—and, on the other hand, if five leeches produce no after-excitement, only two or three should be applied at the next period, and so on.

It is not always, however, that excitement, in any degree, follows these small leechings. The swelling and hardness will be found to diminish after these applications, without any intervening excitement. The same remark, though not to the same extent, is applicable to blisters and the moxa. These sometimes produce their good effects without the intervention of a temporary increase of inflammatory action in the part.

When the leechings cease to be followed by the salutary consequences above described, we are to omit their application for ten, fifteen, or twenty days, when the susceptibility of the parts will be found renewed, as it were, and then we are to reiterate the leeching.

If the leechings do not appear to be productive of the good effects sought for, we must try some other means. If the swelling be only softened, but not reduced in volume, compression may be usefully employed. Next to leechings, however, we must, in general, have recourse to flying blisters. Their effects are analogous to small leechings. They first produce excitement, and subsequently diminution or softening of the tumour. The blisters should be renewed every seven or eight days. When these fail to produce the desired effects, then the moxa is to be employed, on the same principle, and in the same manner as the small leechings and the blisters—that is, about once in seven or eight days. They should not be larger than the size of a five sous piece, in order that the eschars may not interrupt the reiteration of the remedy. Whenever the excitement resulting from the moxa runs too high, then large leechings are to be employed for its reduction. The tumour being softened by the moxa, as by any of the other means, compression, for a time, (eight or ten days,) is to succeed, protecting the eschars by proper dressings. When the disease is very chronic; and when the means already enumerated begin to fail, setons are very serviceable. When they excite too much irritation, they are to be reduced in size, or, this failing to lessen the excitement, leeches and antiphlogistic measures are to be put in force.

Notwithstanding all the means we can employ, it often happens that a certain degree of engorgement remains, especially at the lower part of the joint—and also some effusion in the articulation itself. We need not be anxious about this, as, in the majority of cases, time will remove it. If tardy in its dispersion, mercurial frictions will be serviceable, as also frictions with the ointment of iodine, (hydriodate of potash.) These may be alternated with other stimulating liniments, while the local application of the warm water or vapour bath is to be brought in as an auxiliary of no mean efficacy.

A curious fact is here noticed by M. Lisfranc—namely, that, after the cure of white swelling by the above-mentioned means, the joint gets smaller than before the disease took place, and will continue so for a considerable space of time. The patient should be cautioned to return very gradually to the free exercise of the joint, since relapses are apt to ensue from want of attention to this point. If the disease be in the lower extremity, the patient should go on crutches for one or two months after the symptoms have disappeared. The joint should be kept tightly bandaged for a long time after all other means are discontinued.

M. Lisfranc has repeatedly remarked that, where the white swelling has been situated in the joints of the superior extremities—especially in females, the application of leeches, even in great numbers, and preceded by general blood-letting, has determined congestions in the thoracic organs, and produced difficulty of breathing and palpitations of the heart. In other instances, cerebral congestions have been the result of remedial measures applied to the local complaint, and apoplexy threatened. The local treatment must then be discontinued, and leeches applied to the feet, together with purgatives and diuretics internally. It is also proper to discontinue the local treatment, in the case of females, for a few days previous to each expected menstrual period, lest that process should be interrupted in its course.

The above-described treatment will generally prove successful in the idiopathic white swelling—but the case will be different when we have the *symptomatic* form of the disease to contend with. Here we must attend to the constitutional disorder, of which the white swelling is only a part, or rather an effect. We need not enter upon the constitutional treatment of rheumatism and scrofula in this place; but only remark, that the local treatment, indicated in the preceding pages, will prove serviceable as an auxiliary to the constitutional, in the symptomatic forms of white swelling.

Twenty-three cases are minutely detailed from the practice of Beclard and M. Lisfranc, in La Pitié, illustrative of the various positions laid down in the foregoing paper. These we must pass over, of course, hoping that we have, in a few pages, concentrated a considerable mass of important and useful pathological and therapeutical matter, the result of experience in a public institution, by a man of eminence, talent, and strict veracity.

---

### 2. SLOUGHING ULCERATION.

In the second number, (New Series) of the Medical and Physical Journal, two cases of sloughing ulceration are reported from the practice of Mr. Travers, in St. Thomas's Hospital. The first was the case of a young female lately become abandoned, and consequently addicted to intemperance. She was admitted for a small, irregular, sloughy ulcer on each side of the perineum, of three weeks' duration, for which she had taken no medicine, although her health had suffered severely. She was ordered camphor mixture and nitric ether thrice a day, with an opiate at night, and an opiate application to the sores. In ten or twelve days the sores had become clean and healthy looking. She was now ordered wine and bark ; but at the end of a week, we find the sloughing process had again commenced, implicating the whole of the perineum, and extending to the nates on one side. The sloughs were of a black colour, and threw forth a fetid discharge. The pain was very great, and of a burning kind. The surface of the body was covered with copper-coloured spots—pulse 120—tongue furred—great thirst—constipation. *Venesection—camphor and nitrous ether—opium—port wine—poppy decoction—opiate applications—aperient.* On the 4th day from this time, we find the labia sloughing more rapidly. The undiluted nitric acid was freely applied over the whole wound by means of a pencil-brush, working to the bottom of the slough, which was dense and resisting. Two grains of opium were given and in 20 minutes she was free from pain. Next day, the patient was better, in respect of the local symptoms. The acid application renewed, as was the opium. In two or three days more the slough was thrown off, leaving a florid granulating surface copiously suppurating. The wound is an inch in depth, in some places. The constitutional symptoms are improved—the appetite better—and the febrile irritation less. She improved gradually and very slowly till discharged from the hospital.

The second case was that of a young lad, who came into the hospital for inflamed phymosis and sloughing sore surrounding the extremity of the prepuce. The swelling, tension, inflammation, pain, and sloughing rapidly increased, accompanied by corresponding constitutional irritation. Twelve leeches were applied—the parts fomented—and antimoniated saline aperients given every six hours, with an opiate at night. In three days after this, we find the slough extending, increase of pain,

&c. He was bled to 12 ounces—had the camphor mixture with ether —and an opiate, as usual, at bed-time. The sore remained stationary for three days after the bleeding, and then began again to extend, with increase of pain. A dozen leeches. A grain of opium three times a day. From this time the patient improved rapidly, till he exposed himself one day to cold at a window, when great effusion took place into the integuments of the penis, accompanied by a dark red inflammation, exquisite pain, and anxiety. The strong nitric acid was applied to the parts whence the slough had separated. The pain was very severe, and lasted about 20 minutes, succeeded by perfect ease, and sound sleep. Next day the tumefaction had subsided, the induration and inflammation having abated. A foul sore was now disclosed at the frenum, where alone pain was complained of. Matters again soon took a turn for the worse; the slough gradually spreading till the whole of the original sore was contaminated, with return of pain, tumefaction, and other bad symptoms. In short, the whole prepuce sloughed away, together with a considerable portion of the glans penis, and some part of the parietes of the urethra, so as to form an opening into that canal, an inch from the external orifice. Two days later, nearly the whole glans is reported to be in a sloughy state, as well as a considerable portion of the integuments of the penis, attended by excessive pain and anxiety. The strong nitric acid was again applied, followed by opium reduced to the consistence of cream. Carbonate of ammonia, musk, and opium were exhibited internally. The slough caused by the acid separated in two or three days; but as it fell off, the sloughing process attacked the exposed healthy surface—"its progress not being in the slightest degree impeded by the local application of the medicines." Several other plans were now tried, as the sulphate of quinine—wine—opium —bark—conium—argentum nitratum externally, &c. At length we find the whole penis destroyed, and still the destructive process going on, with agonizing pain night and day. The scrotum was next invaded, and the testes left exposed. It is needless to pursue the diary of this unfortunate victim of illicit pleasures. He died at length, worn out by a series of sufferings, which it is dreadful even to contemplate—and which must have afforded a terrible spectacle, and it is to be hoped, a memorable example for the numerous youths attending the hospital!

The above case presents a melancholy instance of phagedenic ulceration, running on for four months, and resisting the various remedies, local and general, which could be devised. This unfortunate as well as imprudent young man contracted the disease in Swan-Alley—a place notorious for profligacy and debauchery of the most degrading and dangerous kind.

Two cases follow, of "sloughing ulceration" under the care of Mr. Green, at St. Thomas's Hospital, in which the nitric acid was the principal local application. In one case, the termination was favourable—but in the other, (a girl 15 years of age) the labia pudendi, perineum, nates, &c. &c. were destroyed by this terrible disease, and death closed the scene, attended with all the horrors of pain and despair!

Mr. Rose reports two cases, treated at the St. James's Infirmary, of "sloughing ulceration" of the genital organs, in which bleeding, purging, opium, and low diet, led to a fortunate termination. As this is a plan which, in our experience, has succeeded better than any other, in such cases, we shall give the particulars of one of these histories for the benefit of our readers.

*Case.* D. Andrews, 23 years of age, was admitted into the infirmary on the 27th April, having a deep sloughing sore on the inner prepuce, immediately behind the corona glandis, which had almost entirely separated the gland from the extremities of the corpora cavernosa, penetrating into the urethra, the whole sore being covered with a deep slough the edges jagged, and surrounded by a dark red margin. Tongue dry and rough—emaciation—thirst—flushed countenance—hot skin—quick pulse—burning pain in the sore, which had existed for a fortnight, during which he had been taking mercurial pills. *Venesection to 12 ounces—lotion of opium and conium—antimony and sulphate of magnesia every four hours—15 grains of Dover's powder at bed-time.* 28th. Sloughing goes on—blood inflamed. *Repeat the venesection to 14 ounces. Medicines continued.* 29th. Fever continues—no sleep—blood still inflamed—sloughing extends. *Repeat the venesection to 12 ounces, as well as the same remedies.* 30th. Blood less inflamed—has slept some, and is less irritable—sore is easier. *Pergat.* May 1. Some healthy pus on the wound. *Medicine continued.* 4th. Sore has rapidly improved—sloughs are separated—granulations springing up every where. Has left off medicine, and is allowed nourishing diet. From this time every thing went on well, and in three weeks the sore was healed.

The other case was that of a female, the treatment and event being the same as in the former case. The surgical practitioner will choose for himself between the different plans of treatment portrayed in this article. We have stated our own preference—but we may be wrong. Experience must decide.

---

### 3. ON TIGHT BANDAGING IN PHLEGMONOUS ERYSIPELAS, &c.

About ten or eleven years ago, M. Bretonneau, a distinguished physician of Tours, maintained a thesis on this subject in Paris, and was most roughly treated by the professors on that occasion. In the present instance, M. Velpeau, a man of talent and observation, has come forward to maintain the same principles which had been advocated by his friend, M. Bretonneau. Tight bandaging has been long in use for ulcers, œdematous swelling, and other chronic affections, unattended with much pain or acute inflammation :—and M. Velpeau does not seem to know that, some years ago, Dr. Balfour, of Edinburgh, proposed and practised this measure even in *acute* rheumatism. In phlegmonous erysipelas, where the cellular membrane, between the integuments and muscles, and between the muscles themselves becomes disorganized, tight bandaging has been employed in this country, with the view of changing the suppurative into the adhesive inflammation. Free incisions have also been made into the parts, not only to give vent to the purulent matter, but to bring on a different kind of inflammation in the diseased structures. M. Velpeau incidentally alludes to this practice, as pursued by an eminent surgeon of Paris, and brings forward the present paper to advocate the use of tight bandaging, as a measure that will secure all the advantages of free incisions, without the pain or formidable appearance attendant on the operation by the scalpel.

Ten cases are selected by M. Velpeau from the practice of "*l'Hôpital de la Faculté*," for the elucidation of his subject; consequently the cases are authentic, being under the eye of many spectators, who could readily detect any misrepresentation. We shall give a brief account of a few of these cases.

*Case* 1. This was a young woman who entered the hospital for the removal of a steatomatous tumour from the ham, which was done a few days afterwards, without difficulty, and without any untoward symptom, until the 8th day, when fever was kindled up, and inflammation extended about the wound. It took on an erysipelatous character, and soon reached within a few inches of the groin. Bleeding, leeching, &c. were employed, but the fever persisted, and "every thing indicated the establishment of suppurative inflammation in the cellular membrane of the thigh." It was even feared that matter had actually formed. The surgeon (M. Bougou) now authorised M. Velpeau to employ his compressive bandages, which were accordingly applied to the thigh—one between the groin and the erysipelas —the other above the calf of the leg. Compresses moderately tight were also applied between these two points. In the course of the evening the general symptoms of excitement had almost entirely subsided—the pain of the limb was greatly mitigated—the inflammation had ceased to extend—and, in short, the erysipelas disappeared in two or three days more.

*Case* 2. A man, 45 years of age, came into the hospital, for the cure of an ulcer on the shin of 18 months' standing. The healing of the ulcer was far advanced, when, for the purpose of accelerating the cure, the man was bled from the arm. There quickly supervened considerable fever and constitutional disturbance, followed by an erysipelatous inflammation on the ulcered leg, extending from the heel to near the knee. Red streaks were also seen extending up the thigh to the groin. To this part a large blister was applied. The fever and inflammation of the thigh subsided; but the leg became greatly swelled, and all the medical officers believed that suppurative inflammation was going on in the cellular membrane of the limb. A bandage was applied methodically by M. Velpeau, from the heel to the knee. During the first hour the pain was rather increased, but afterwards diminished gradually, and had nearly ceased by the evening. Next day, the redness and swelling had greatly subsided, so much so, that had not the constitutional symptoms also subsided, *pari passu*, with the local, the medical attendants would have apprehended a metastasis. This did not happen, and the patient was well in a few days.

M. Velpeau here asks whether the constitutional disturbance, which became developed in this case immediately after the venesection, could have been owing to that operation, or a mere coincidence? We think there can be little doubt that the *latter* was the case. It was very improbable that an erysipelas of the leg could be suddenly set up by a bleeding from the arm. The explosion was ready to take place in the constitution, and it is not quite impossible that the venesection accelerated the event. The good effects of tight bandaging, in this instance, are certainly apparent, if not real.

*Case* 3. A woman, 46 years of age, had the right mamma amputated for scirrhus of that part. During eight days afterwards she suffered much from pain in the right arm, which was not attended to, till the member began to swell and exhibit a shining appearance with redness. Leeches were applied in great numbers, and renewed every two or three days. The pain was diminished, but the redness and swelling continued, and even increased. It was then determined to use compression. A tight glove was applied to the fingers and hand, while a bandage went spirally up the arm to the arm-pit. The pain was entirely removed in one day—and in two days the swelling had nearly disappeared. In the course of five days the arm was in such a good condition that the dressing was confided to the common dressers of the ward, and the patient was soon cured.

This swelling of the arm is not an uncommon consequence of removal of the female mamma, and often ends in extensive abscesses or troublesome erysipelas. Compression is undoubtedly a useful measure in such cases; but few would think of applying it while acute inflammation was going forward in the part.

*Case* 4. A young man, 23 years of age, of strong constitution apparently, entered the hospital on the 6th January, 1826, for the cure of several small ulcers, surrounded by considerable swelling and hardness, on the left leg. These had been of two years' standing. M. Roux ascertained that various openings on the surface conducted by different fistulous canals to the cellular membrane within the tendo achillis. Several incisions were made; and sinuses laid open. At first the appearances of things were bettered, but after a time, the glands of the groin swelled, and the whole limb became affected with erysipelatous inflammation, and was very painful. Constitutional fever and irritation supervened. The external erysipelas was dispersed, but great swelling of the limb continued, with every symptom of deep-seated suppurative inflammation. At this time the compressive bandage and adhesive plaster were applied, in the manner recommended by Baynton, and the very best effects followed. The size of the limb was soon reduced, the ulcers cicatrized, and the deep-seated abscesses healed.

The above cases are sufficient specimens of a practice which is much more common in England than on the Continent, although M. Roux, after his journey to this country, naturalized, as it were, the Bayntonian method of treating ulcerated legs, in France.—*Archives, Juin,* 1826.

### 4. FOLLICULAR ULCERATION.*

In various parts of this Journal, we have endeavoured to draw the attention of our brethren in this country to the state of the mucous membrane of the primæ viæ, in fevers and other diseases; and we are glad to find that medical men are now in the habit of examining this important tissue much more scrupulously than formerly. In our ninth Number, we gave some account of the researches of Dr. Bretonneau of Tours, and Dr. Troillet of Paris, in respect to the formation, progress, and cicatrization of intestinal ulcerations. Our countryman, Dr. Hewett, has not been idle since his appointment to St. George's Hospital; and we confidently anticipate many useful investigations from this intelligent and well-informed physician, during his official labours at the above-mentioned establishment. In our cotemporary for August, Dr. H. has detailed ten cases of follicular ulceration, observed in fever patients, in the wards of St. George's Hospital. We are not able to afford space for any of these cases, as they cannot be abridged without detriment; but we shall endeavour to give an abstract of those pathological observations which Dr. Hewett has appended to the cases themselves.

His object is to shew the origin and progress of the disorganizing process of follicular ulceration, "during the course of the idiopathic fever," without at all maintaining that this is the cause of the fever.

"A reference to the above post-mortem examinations will shew that the disease seems at first to be limited to the mucous follicles, affecting both the glandulæ aggregatæ and solitariæ, more especially the former, where distributed over the ileo-cæcal portion of the intestines;—that their orifices may be seen plugged up with a dark and morbidly inspissated secretion;—that this secretion continuing, and being pent up within the follicle, in consequence of the obstruction of its orifice, tumefaction of the gland necessarily ensues, as in the sebaceous follicles in acne punctata; that these mucous glands may be seen in various stages of enlargement,—at first only slightly elevating the mucous membrane, and, by their protrusion of it, presenting an uneven granulated surface, like that of dried seal-skin;—that, in most instances the incipient tumefaction of the follicles, and in many instances their ulceration, takes place without any increased vascularity of the neighbouring surface;—that the disorganising process, by affecting many of the contiguous mucous glands, forms ulcers of the size of a sixpence or a shilling, or even larger in some instances, without any increased vascularity, but more generally with slight redness and considerable thickening of the mucous membrane immediately surrounding the margin of the ulcer;—that the margins of the ulcer frequently appear, at least in the early stages, puckered, jagged, or fringed;—that the base of such ulcers often exhibits a rough irregular surface, with little projecting masses of enlarged and sloughing follicles, or presents a sloughy ash-coloured appearance, resembling that of chancre, or displays a dirty-yellow hue, from the

* Dr. Cornwallis Hewett, Physician to St. George's Hospital. Med. Journal, Aug. 1826.

bilious-coloured feculent matter adhering to it;—that the ulceration seems to commence sometimes at the apex of the tumefied gland, sometimes in the membrane protruded, and thus forced into ulceration by it;—that the work of disorganization seems to be effected partly by the ulcerative, partly by the sloughing process;—that each individual follicle gradually sloughs off, in proportion as its neighbouring membrane is eroded by ulceration;—that this erosion extends gradually downwards, from the mucous to the muscular and peritoneal coats, sometimes, though rarely, perforating the latter, and then rapidly exciting extensive peritonitis, by the escape of foreign matter into the cavity of the abdomen;—that this perforation of the peritoneal coat is sometimes prevented, by the effusion of lymph from its surface, external to the ulcer, causing agglutination of it to some of the neighbouring convolutions of the intestines, or to the omentum;—that the mesenteric glands, communicating with such ulcers, are generally found increased in size and vascularity; and sometimes, when cut into, resembling, in colour and consistence, the pulp of a Morella cherry, or even that of a red cherry." 109.

The symptoms attending the recovery of some cases (hereafter to be described) seem to Dr. H. to warrant the belief that such ulcers had existed, but had subsequently healed.* A conclusive proof of this last position was afforded Dr. Hewett in the dissection of a sailor who, fourteen months previously, had suffered severely from dysentery on the Coast of Africa. From this he slowly recovered, but died in St. George's Hospital of a disease quite unconnected with the dysenteric affection. On dissection, the mucous membrane, from two or three inches above the ileo-cæcal valve along the whole course of the colon and rectum, appeared spangled with circular or irregularly-oval scars of former ulcers. In some, however, the process of reparation had not been completed—and along the base and margin of these healing ulcers, there was distinctly seen a thin film of yellowish white coagulable lymph, proceeding apparently to fill up the ulcerated cavity.

"In many instances it had not yet reached the level of the adjoining surface; in others, it had: in the latter, the regeneration of the surface seemed complete, the lymph having become so intimately blended with the neighbouring membrane as to be scarcely distinguishable, except by the glistening and spangled appearance above mentioned. In the former, the process of reparation seemed to be gradually advancing: in these, the neighbouring edge of the mucous membrane (which had probably been previously thickened and fringed,) appeared to be gradually subsiding, contracting and bevelling itself down so as to favour the progress of the cicatrization. Where the breach of surface had been completely repaired, there was no increased vascularity; and only a very faint appearance of it in the ulcers undergoing the healing process; the degree of redness seeming to vary according to the exigencies of

* See our review of Dr. Latham's work on the Diseases of the Penitentiary Prisoners—and also Dr. Troillet's paper on the Cicatrization of Intestinal Ulcers, No. 9 of this series, p. 192.

each individual ulcer, being more or less, in proportion as more or less lymph was required for the filling-up of the ulcer, and gradually becoming evanescent, as the continuity of the surface was re-established.

"The healing of these ulcers appeared to me to be effected, not, as in external ulcers, by any visible growth of granulations, but by a process analogous to that which is displayed in the reparation of ulcers of the cornea, by the deposition of lymph." 110.

Those, indeed, who have practised in climates where dysentery prevails, must have had numerous opportunities of seeing these remains of former ulcerations, when their patients happened afterwards to die of other diseases. But they have seldom recorded with minuteness the appearances which these intestinal cicatrices present.

Dr. Hewett observes that, in most of the cases of follicular ulceration in idiopathic fever, the seat of the ulcers will be indicated, upon opening the abdomen, by some deviation from the natural transparency of the peritoneum—"either by small opake patches of silvery whiteness, or by apparently attenuated spots, occurring in discoloured portions of the intestines." It not unfrequently happens, however, that there are no particular appearances of the peritoneum to indicate the existence of these follicular ulcers—hence they have generally been overlooked.

Another morbid appearance which our author has occasionally detected in the mucous membrane of the intestines, in those who died of protracted fever, when abdominal derangement had entirely subsided, or was not prominent, was as follows:—light buff-coloured patches of various forms and sizes, without elevated borders, but with a well-defined outline, marked by the sudden difference of colour between them and the adjoining surface. The inclosed area is traversed by numerous thin seams or septa, forming, by their interlacement, minute cellules, the base of which appears to be either the denuded peritoneum, or a nearly transparent film of lymph, deposited upon that membrane.

Dr. Hewett bears testimony to the general accuracy of M. Bretonneau's description of intestinal ulcerations, as given by us in our July number of this Journal—and promises, in the next paper, an attempt to ascertain the period of idiopathic fever, at which this follicular ulceration of the intestines usually appears—together with a plan of treatment suitable to these diseased conditions. The following passage, should it ever meet the eye of Broussais, will make his hair stand on end, "like quills upon the fretful porcupine."

"At present, I would only beg permission to state, that these pathological views afford an additional and powerful argument for active and persevering purgation by effective doses of calomel, combined with any of the usual auxiliary purgatives, in the early stage of fever. Its power of preventing follicular ulceration, seems readily explicable on the following principles:—The preceding investigations appear to have established that the first step towards follicular ulceration is obstruction of the orifice of the gland by its own morbidly-inspissated secretion; the next step being distention, from the continuance and accumulation within it of this secretion, and subsequently ulceration. If, then, at this period effective purgatives be employed, they will clear out the obstructed orifices, and thus anticipate the distention and subsequent ulceration of the follicles." 114.

The disciples of Broussais will draw a conclusion the very reverse of this, and accuse the purgative plan of Dr. Hewett as one of the principal causes of the very ulceration which it is meant to prevent! Such are the different views entertained on the north and south sides of "La Manche!"

Since the above was written, Dr. Hewett was kind enough to shew us an exquisite specimen of this follicular ulceration, (or, as the French pathologist terms it, "pustular enteritis,") which was presented in the intestines of a female who died of fever. The various stages of the disease, from a small rounded prominence, not larger than a pin's head, to a broad streak of ulceration, leaving only the peritoneal covering entire, were most distinctly marked in this case—the disease occupying many feet of the small intestines.

We have no doubt that this disease is of frequent occurrence, and that it has been often overlooked, from not examining, with care, the mucous surface of the bowels. The writings of Bretonneau and Dr. Hewett will, doubtless, excite attention to the subject in this country.

---

### 5. ON FRACTURES OF THE SPINE. BY M. LISFRANC.

The general fatality that attends these accidents, and the difficulty of managing them, has led to a reluctance in studying them. This should not be the case. Fortunately the spinal column is so constructed and protected as to be rarely fractured—and still more rarely luxated, except at the summit of the chain. The fracture may be in the spinous or transverse processes, or in the body of the vertebra. The signs of this formidable accident are not always unequivocal, and are sometimes very much the same with those which attend certain natural dispositions of the spinal column. In these doubtful cases the patient should be placed in the horizontal posture, and the spinous processes carefully examined. Great care, however, should be taken in making these examinations, as a very slight motion or twist might produce the most irremediable mischief. M. Lisfranc has ascertained that the stethoscope applied to the ribs, or even to the sinciput, will give intimation of such fractures, by the grating noise produced. In fractures of the spine, the spinous processes are sometimes elevated, sometimes depressed below their natural level. No dependence is to be placed on the degree of pain of which the patient complains. The most certain diagnostic symptom was paralysis—especially loss of *motion*, for the loss of *sensibility* in the parts below the fracture is a much less common phenomenon.

The bladder and rectum more frequently feel the effects of this paralysis than any other internal organs. On this account the catheter should be introduced at least twice a day. If there are liquid fæces in the rectum they will pass off involuntarily—if solid, they will be retained. Sometimes, when the spinal marrow is slightly injured, inflammation will arise—and instead of paralysis, we shall have convulsive movements, subsultus tendinum, or even tetanus. Meteorism, or tumefaction of the abdomen, is among the most common consequences of spinal fracture. When the patient dies in ten or fifteen days after the accident, the intestines, rectum, and bladder generally present evident traces of inflammation. In fact, the urine and fæces remaining in contact with their respective mucous surfaces (where the organic sensibility is not destroyed, in consequence of nervous influence being derived from the great sympathetic) occasion irritation, and ultimately inflammation. This irritation and inflammation may be recognized during life, by the morbid secretions produced in the bladder and intestines. The thick glairy mucus secreted in the bladder tends greatly to prevent the free discharge of the urine, and requires sometimes the introduction of warm watery fluids for its dilution. The urine, in such cases, has always a strong ammoniacal odour. In many cases death is not produced by the paralysis, but by these consecutive inflammations—hence the special care which we should take to obviate these consequences, and thus give the patient every chance for his life.

In all shocks of the spinal column, whether there be fracture or not, we have to apprehend injury on the part of spinal marrow—primitive or secondary. In the first instance we sometimes observe no particular effects for several days—then the consequences of effusion, inflammation, &c. begin to shew themselves. Thus, a man in the wards of M. Dupuytren had received a contusion of the spinal column by a fall. At first there was only a slight degree of paralysis of the lower extremities, which disappeared in a few days. At the end of ten days the paralysis returned, but in a much stronger degree. The symptoms increased, and death ensued. On dissection, no fracture could be discovered. There was merely an extravasation of sero-sanguineous fluid at the lower part of the dorsal region of the spinal canal. In the same wards, and under the eye of Lisfranc, the following case occurred. A woman slipped down a flight of steps, and struck her spine (about the lower cervical and upper dorsal vertebræ) against one of them. She was brought to the Hotel Dieu, with paralysis of the lower extremities, rectum, bladder—together with loss of sensibility in the integuments of the abdomen and thorax. She breathed only by means of the diaphragm. From day to day the paralysis made progress. The arms got benumbed, and the march of mischief in the spinal canal could be traced up to the origin of the phrenic nerves. The patient died in a state of asphyxia. On dissection, the body of the seventh cervical vertebra was found to be fractured, together with a rent in the spinal marrow opposite the fracture. From this point up to the origins of the phrenic nerves, there was evidence of acute inflammation, and also of softening of the medullary mass.

The *prognosis* in fractures of the spinal column must be always gloomy. When death occurs after the 10th, 15th, or 20th day, it is almost always owing to consecutive inflammation of the spinal marrow. Some patients have gone on to the 40th day—others have lived paralytic more than six months—and some have survived and continued in a state of paralysis. In these cases, the members waste—and when eschars form, they are very slow in their progress, being generally of a dry nature, the cicatrization being very tedious.

*Treatment.* Are we to attempt the reduction of fractured bones in the spine, and keep them in coaptation? If the fracture be unaccompanied by paralysis, we should be very cautious how we attempt reduction, however widely the fragments may be separated from each other. In such attempt we may injure the spinal marrow, and thus run into the very danger we wish to avoid. In the mean time, it is evident that we ought to fix the patient in such a position that he may not himself execute any movements likely to injure the medulla spinalis. Two fatal examples of the disregard of this precept were witnessed by M. Lisfranc. If a severe degree of paralysis accompanies the fracture; then, indeed, we are bound to reduce it, if possible, since we can hardly increase, and may, perhaps, lessen the mischief. "When the reduction is difficult," says our author, "the English have proposed to cut down upon the bones and lay them bare, in order that the depressed parts may be seen and elevated. In some cases they have trepanned the spine with success." We should be much obliged to M. Lisfranc to point out these cases of success.

Our next object is, of course, to prevent, or to keep down inflammation, by general and local bleeding. Leeches, in great number, should be applied to the spine, even when there are symptoms of concussion, with coma, &c. provided the pulse be strong and regular. By these means M. Lisfranc has been successful in several cases of fractured spine. The bladder and bowels are to be carefully attended to, for the reasons pointed out above. When the paralysis supervenes some time after the accident, and when it cannot be attributed to osseous compression or wound of the spinal marrow, we must then conclude that it is owing to inflammation or extravasation, in which cases we have to trust almost entirely to bleeding and counter-irritation. Such are the general indications drawn from numerous facts, some of which we shall lay before our readers in a future number.—*Rev. Med. Nov.* 1825.

---

### 6. MR. GUTHRIE ON PHLEBITIS, &C.

*Phlebitis after Amputation.* Mr. Guthrie [Med. and Phys. Journal, No. 1,] remarks, that phlebitis is one of the most fatal consequences of amputation, and occurs most frequently where the patient has been previously in an irritable or suffering state. The inflammation is of two kinds—adhesive, or healthy—and irritative, or unhealthy. The *former* often passes unobserved, or, if observed, is easily cured. The *latter* is generally fatal. It is, Mr. Guthrie thinks, the most common cause of death after amputation. When it is about to take place, the pulse quickens, the stomach sickens, vomiting of bilious matters occurs, and the usual symptoms of fever follow, with rigors and remissions. The patient emaciates rapidly—the skin becomes tinged yellow, and often covered with perspiration—bowels irregular. The pulse becomes quicker, weaker, and more irritable—and the patient gradually sinks. Or, perhaps, he may rally a little, for a time, and think himself getting better, whilst the deterioration of his appearance is evident to the surgeon; and a new accession of fever closes the scene. There is often no more pain or suffering about the stump than usual, nor any remarkable tenderness in the course of the vessels. In the case which Mr. G. relates in illustration, the femoral vein, as high as Poupart's ligament, on the amputated side, was frequently examined by pressure, and also the iliac, but no uneasiness was complained of. The evolution of heat was considerable. "The sudden and great enlargement of the whole of the superficial veins was early very conspicuous, which, with the severity of pain experienced at all times, and augmented to agony on the slightest motion, were the only perceptible differences, and those only in degree, to be observed between it and a case of phlegmasia dolens, either by comparison with what I recollected or recollect of cases previously or subsequently seen." It was also the only instance out of a great number of cases, in which the femoral veins of the opposite side became affected after amputation. Mr. G. has seen the opposite iliacs frequently implicated, but never remembers to have seen the inflammation proceed lower. In few can the inflammation be traced higher than the diaphragm—indeed, the patient usually dies before it passes the junction of the emulgent veins.

Mr. Guthrie appears to consider Dr. Davis's theory of phlegmasia dolens as essentially correct—the venous inflammation, in this disease, being most frequently of the healthy or adhesive kind; whereas, in amputation, it is of the unhealthy, or erysipelatous kind. But Mr. G. does not coincide with Dr. Davis in the *cause* of the phlebitis in phlegmatia dolens, (pressure of the uterus on the veins,) nor in the locality of its origin. "I would suggest," says he, "for the future, the propriety of tracing the veins from the common iliac of the affected side down to the uterus; and when attention is particularly directed to this point, I have little doubt but the inflammation of the veins will be found to have begun at the uterus, and to have ascended along a continuous surface, until it implicated the veins of the extremity." This, of course, is only conjecture on the part of Mr. Guthrie; and neither it nor the dissections and reasonings of Dr. Davis have yet convinced us that the *primary* cause of phlegmatia dolens is inflammation of the veins. At the same time we are ready to admit that Dr. Davis has made out a very plausible case, and has hit upon a very ingenious theory. Future investigations are necessary to shew how far it explains the phenomena of this curious affection. We shall give a very brief outline of the case published by Mr. Guthrie.

Jane Strangemore was admitted into the Westminster Hospital for an elastic swelling of the knee-joint, measuring 27 inches in girt. It was of five months standing, and was attended by severe paroxysms of pain during that period. Amputation just below the trochanter was performed by Mr. G. three days afterwards. Next day the pulse rose, and the stomach became irritable. Proper medicines were administered, and she seemed better. On the 4th day the stump was dressed and looked well—pulse 120. On the 6th day there was much pain in the stump, which was caused by change of dressings. On the 12th day every thing was promising, and the patient ate meat with an appetite. On the 13th day fever set in, and pain was complained of in the other leg and thigh. She was well purged, and the member fomented. 14th day, pulse 130—furred tongue—bilious vomiting—the pain in the thigh, extending up to the groin and down to the heel, intolerable—thigh and leg much swelled, tender to the touch, elastic, and not red or œdematous. Mr. G. pronounced the disease to be phlebitis. She lived 17 days after this, sometimes better, sometimes worse; but always emaciating, and the pulse ranging from 126 to 136, the appetite generally good. At the period of her death the limb had every where diminished in size, except at the groin, where it was circumscribed, resembling a chronic abscess approaching the surface.

On dissection, the vein was open on the face of the stump, and in a sloughy state—the inside of the vessel as high as Poupart's ligament, bore marks of inflammation, but of the adhesive kind. Above that point it appeared of the erysipelatous kind, and had gone on to suppuration, the vein being filled with purulent matter, lymph, and blood. These appearances extended to the cava beyond the diaphragm, with some traces of inflammation almost to the heart. This disease had passed along the right internal iliac and its branches, and descended along the left iliac vein, affecting its branches in the pelvis to the uterus, and along the limb to the sole of the foot. At the left groin, the iliac vein becoming femoral, was distended with pus of good quality. The viscera were healthy.

How far this disease will be admitted as identical with the phlegmatia dolens of puerperal women, we leave to our obstetrical brethren to decide. For our own parts, we have seen six well marked cases of the latter, and we do not admit the identity. We have not room, however, to state the points in which they differ.

---

### 7. ŒSOPHAGITIS—HYDROPHOBIA.*

The following case proves one of two things—either that the bite of a dog (which was not proved to be mad) could be followed two years afterwards by symptoms of hydrophobia; or, what is much more likely, that hydrophobia may result from other causes than the bite of a rabid animal.

---

* Ch. Pfeuser. Senior Physician to the General Hospital of Bamberg.—*Heid. Klin. Annal.* 1825.

*Case.* J. Kneiss, aged 48 years, of sanguineous temperament, and in excellent health, received a slight scratch from a dog, that did not evince, before or after, any appearance of hydrophobia. He took no notice of the accident. Nearly two years after this, having drank freely of cold liquids while heated by work, in the month of May, he soon experienced a difficulty of swallowing fluids, a sense of weight in his limbs, and a feeling of intense cold throughout his body. He took some trifling medicines, and on the third day there was violent fever, great thirst, and such a spasmodic affection of all the parts about the œsophagus and larynx, on the attempt to swallow liquids, that he fell into a kind of deliquium. This was reproduced by each succeeding attempt. On being received into the hospital of Bamberg, he presented, besides the foregoing symptoms, a remarkable velocity in all his movements, a loquacity of speech, and a furious expression in his countenance. The pulse was quick and full, the action of the heart precipitous, and sometimes suspended, skin hot and dry, thirst considerable, tongue coated, urine scanty, red, and turbid ; deglutition of solids easy. He was bled to 32 ounces, and had a laxative enema. The ensuing night was spent in dreadful agitation, with occasional delirium, convulsive movements, and fits of lypothymia. The pyrexia now ceased for a time, and calomel with belladonna, in small doses, were prescribed, while mercurial frictions with the same were applied along the spinal column. The thirst soon afterwards became intense, the pupils dilated, and a frothy mucus was ejected from the mouth. These symptoms continued for two days more, and the sight of water or any other liquid produced horrible convulsions. The patient complained of intense pain in the region of the stomach, and the tongue seemed inflamed. Eight ounces of blood from the jugular vein, and 20 leeches applied to the head. Ice was also applied to this part, a blister to the nape of the neck, and sinapisms to the lower extremities. In the evening, a severe paroxysm of agitation, and 32 ounces of blood were abstracted. A complete remission of all the symptoms followed, the patient was calm and collected, full of hope, and swallowed whatever medicines were offered to him. This state of tranquillity lasted all the day, and in the beginning of the night he had three hours' calm sleep. From this period he fell into a state of extreme apathy, stupor, and complete prostration of physical and intellectual forces. He died in a few hours afterwards.

*Dissection.* The cerebral substance was extremely soft—spinal marrow sound—salivary glands very much swelled—some gangrenous eschars on the surface of the pharynx and larynx, the vessels of which were extremely injected—thyroid gland enlarged, changed in colour, soft, and full of thin black blood—œsophagus inflamed throughout its whole extent, and presenting some spots of gangrene—the diaphragm in the same condition—mucous membrane of the stomach and duodenum very red—pancreas in the same state as the salivary glands.

This is a curious case ; and, we think, there can be little doubt that the ingurgitation of cold liquids, when the body was heated, produced the inflammation in the mucous membrane of the parts above-mentioned; and that this inflammation, tending so rapidly to gangrene, gave evolution to the whole of the phenomena described. The more we study the physiology and pathology of these mucous membranes, the more we shall be astonished at the overwhelming influence which irritation of their nerves exerts over the whole of our physical and intellectual functions. This study is yet in its infancy, and it presents the widest, as well as the richest field for genius and talent to cultivate.

---

### 8. ACUTE RHEUMATISM OF THE JOINTS.

It appears from the Hospital Reports of our cotemporary, the Medical and Physical Journal, that Dr. Chambers, of St. George's, is in the habit of distinguishing rheumatism affecting the synovial membrane and bursæ of the tendons, from that which affects all the neighbouring parts in the first instance. These varieties, however, pass into each other, though they more generally run a distinct course. The treatment of the *diffuse* form, consists in the administration of calomel and opium—generally ten grains of the former and two of the latter, every night, or night and morning, with a daily black dose to clear the bowels. As soon as the mouth becomes affected, the symptoms usually subside, and the medicine is discontinued. This plan has also been pursued by Dr. Macleod, with great success, for several years past. The treatment of acute, and we might say of chronic rheumatism, by calomel and opium, is employed by many practitioners, and is that which we have had recourse to for twenty years past. But colchicum and occasional bleeding are very useful auxiliaries, and often abridge the quantity of mercury which would be otherwise necessary. These auxiliaries also prevent the necessity, in many instances, of affecting the mouth at all—a circumstance of considerable importance in private practice, though not so much so in that of an hospital.

In the August Number of the same Journal, Dr. Chambers has published some cases illustrative of rheumatism affecting the synovial membranes, and in which the treatment is somewhat different from that described above. A single case will exemplify the nature and treatment of synovial rheumatism, according to the views of the reporter.

*Case.* Susan Church, aged 17 years, has been ill nine months with occasional swellings of synovial membranes, the urine being always thick at the time of the attacks. She has now (June 14, 1826) pain and swelling occupying the extensor tendons of both hands, particularly the left, with effusion into the sheaths of the tendons, and into some of the joints of the fingers. Pulse 100—skin cool—tongue slightly furred—bowels natural. *A draught of pimento water, with* 20 *minims of vinum colchici every six hours. Low diet.* 15*th.* Knees much distended—hands better. *Ten leeches* to the left knee—spirituous lotion to the parts affected. The colchicum continued. 26*th.* The medicines have been continued, and the leeches four times repeated. The knee is much better. From this time, she gradually recovered, and was discharged cured on the 3d of July.

The treatment, in short, which has been adopted for this kind of rheumatism, consists in leeching, evaporating lotions—colchicum—calomel, and other cathartic medicines—with occasional blisters. This plan we believe is the one most commonly put in force by practitioners in general—and is quite unobjectionable.

---

### 9. DR. BLAND'S HOSPITAL REPORTS.

In the Nouvelle Bibliothéque for 1826, Dr. Bland has published some of the principal cases which occurred in the Hôpital de Beaucaire, of which he is physician. We shall notice briefly here such as present any features of interest or importance.

*Case* 1. Denis Faucon, æt. 39, a gardener by trade, had been affected for upwards of two years, with severe attacks of gastric irritation, which latterly had increased much in intensity. July 2, 1825, he applied to Dr. B. with the following symptoms. Extreme emaciation, dejection of mind, sleep broken, tongue red, and its papillæ elevated, some appetite, a feeling of weight and oppression in the region of the pylorus, changing about an hour after taking food into insupportable pain, attended with nausea, and vomiting, generally of a liquid matter. It is remarkable, that after taking a full meal of animal food, these symptoms would not make their appearance, while, on the contrary, on eating vegetables, or any light food, the pain, vomiting, &c. would be sure to occur.

Supposing the case to be scirrhus of the pylorus with chronic gastritis, Dr. Bland prescribed one grain of extr. cicut. three times a day, with some other remedies of no importance.

On the 5th of July, in the middle of the night, during a fit of vomiting, the patient expelled a hard, black body, which, on examination, proved to be a peach-stone, its roughnesses rounded down apparently by attrition. The man now said that several years before the first appearance of the disease, he was fequently in the habit of going to sleep with a peach-stone in his mouth.

From this time the disease began to be mitigated, though it did not quite disappear till the end of January, when Faucon regained his health and former enbonpoint.

*Remarks*. The length of time the foreign body remained in the stomach without being suspected; the resemblance which the symptoms bore to those of schirrhus of the pylorus; and finally the non-appearance of the regular paroxysm on eating a quantity of animal food, render this case not only uncommon, but very interesting. There is this difference between the history of this affection, and that of cancer of the stomach, that in the latter, any thing which calls for great action in the organ, such as a full meal, so far from affording relief, as happened in this instance, causes great aggravation of the sufferings. The relief experienced when the stomach was distended, no doubt arose from the viscus being so prevented from coming in contact with, and acting on, the irritating body.

*Case* 2. Jean Baumelle, æt. 59, entered the hospital on the 2d July, 1825, presenting the following symptoms; dull, bearing down pain in the hypogastrium; great difficulty and straining in making water; the urine fetid, and always preceded by a quantity of muco-purulent matter, mingled at times with clots of blood; occasional hæmaturia. On introducing the finger into the rectum, the bladder was felt thicker and more resisting than natural. The stools were healthy, the emaciation and debility excessive.

Iodine was the remedy employed with the effect of gradually lessening the difficulty of passing the urine as well as the quantity of muco-purulent matter, which accompanied it. This treatment was continued till the 15th of September, when it was discontinued, the patient being nearly well.

*Remarks.* Dr. Bland remarks, that in his hands the hydriodate of potass has been successful in arresting chronic inflammation of the mucous membrane of the nose and bladder: we hope that the medicine will prove equally efficacious in the hands of others.

Here follow four cases of tic-douloureux of the sub-orbital and dental divisions of the fifth pair of nerves. The gist of our author's observations is this, that where the neuralgia was *intermittent*, the sulphate of quinine was effectual in stopping the disease; where, on the contrary, the paroxysms were only *remittent*, the quinine was found ineffectual, and the extract of henbane, valerian, and oxide of zinc, succeeded in checking them.

Three cases of carbuncle are detailed, one of them fatal. The first appeared on the right cheek of a man of upwards of fifty years of age, of a strong constitution, and residing in an unhealthy neighbourhood. M. Linnée, the surgeon of the hospital, having made a crucial incision into the pustule, placed in the wound a quantity of caustic potass. The disease, however, spread rapidly, and Dr. Bland being called in consultation, a second cauterization was practised on the subjacent cellular tissue, fomentations were applied, and the man did well.

The next patient was not so fortunate. He resided in the country, was apparently healthy, and was forty years of age; the carbuncle was seated near the sternal extremity of the left clavicle, cauterization was freely employed, but in two days he sunk.

The third case was that of a girl, ætatis 12; the anthrax was here situate upon the left upper eyelid; leeches, cataplasms, and strict diet were the means employed. The girl recovered.

Our author remarks, and we entirely agree with him, that where there is little action, where the disease seems indolent, and where the gangrenous parts are not divided from the healthy by a well-defined inflammatory circle, cauterization is the plan indicated. Where, on the contrary, there are severe pain, febrile heat and strong pulse, the antiphlogistic treatment will be found the best.

---

### 10. MR. SHAW ON HERNIA.

There is no surgical disease which presents a greater variety of appearances and phenomena modifying the treatment than hernia. Mr. Shaw, in his hospital report from the Middlesex, has stated a curious case of a man brought into that institution in the middle of the night, with a large scrotal hernia, which had come down the preceding afternoon. The disease was of ten years' standing, but had been reducible by the patient himself. He complained of much tenderness near the neck of the tumour, and pain darting towards the back. Various attempts by the taxis were made—venesection—ice—stimulating injections, &c. but without success. The warm bath and bleeding to syncope, did not succeed. Next day the tumour was of immense size, extending more than half-way down the thigh, with the testicle projecting at the lower part, and the skin dragged off the penis. The sac of the tumour was tense, but the skin was loose. The principal seat of pain was opposite to the ring, where the tumour abruptly ended. At first it was thought that the great size of the tumour was owing to air in the gut; but this was disproved by the operation, which was performed by Mr. Shaw. The hernia being unusually large, it was determined to make a small incision only, so as to expose the ring, and not to divide the neck of the sac, unless it should be found necessary. When the division of the ring was made, the inferior edge of the internal oblique muscle, with a fascia, and some fibres of the cremastic muscle, presented themselves. There was obviously great relaxation of the neck of the hernia produced by the division of the external abdominal ring, and an attempt at reduction was made, but given over, as it appeared that the neck of the sac itself produced a degree of stricture. This part was, therefore, pinched up and cautiously opened to the extent of an inch and a half, when the finger ascertained that the stricture was removed. In the operation of reducing the hernia now, a jet of serum issued from the wound, and continued to flow to the extent of more than a pint. The tumour was, in consequence, greatly reduced in size, there being only left a small knuckle of intestine which was easily returned. The lips of the wound were brought together by ligatures and adhesive plaster. An anodyne draught was exhibited. The patient did well, and on the 10th day after the operation, was free from complaint. *Medical Journal No.* 1.

In the same Journal, Mr. Boyle, surgeon of the Middlesex Infirmary, has related a case which is of very rare occurrence—namely, where the operation for hernia was twice performed in the same place. The patient had been operated on four years previously, and had worn a truss till shortly before the present accident. The taxis was tried, but without effect, and Mr. Boyle proceeded to the operation. When the sac was laid open, a table-spoonful of serous fluid escaped. The stricture was found at the inner margin of the tendon of the external oblique muscle, and was removed by a gently conducted sawing motion of a probe-pointed bistoury. Every part of the protruded intestine had a livid appearance. It was returned—the wound united by two stitches—and the usual dressings applied. The bowels were soon opened after the operation, and in six days the parts were completely united.

---

### 11. OBSERVATIONS ON URETHRAL STRICTURE.

It is well known that a rigor, succeeded by more or less of re-action, occasionally follows the introduction of a bougie into the urethra. When this accident does occur, it is generally after the first or the second introduction of the instrument. There is seldom more than the one paroxysm produced, and the patient is quite well in a day or two. But sometimes things turn out otherwise, and the patient continues for several days in a feverish and uncomfortable state, with head-ache, white tongue, thirst, and loss of appetite. This state, in some instances, has been renewed every time the bougie has been introduced, and the operation is obliged to be discontinued, lest the constitution should suffer more than the local complaint might gain. An eminent surgeon of this metropolis was thus circumstanced lately with one of his patients, who was an elderly gentleman, having stricture in the urethra, and an enlargement of the prostate gland. When he was on the point of giving up the attempt at using the bougie, he observed that the rigor did not come on immediately after the introduction of the instrument, but directly on making water for the first time after the operation. This observation induced him to try the experiment of allowing a hollow bougie to remain for some time in the urethra, so that the water might drain off, without touching the irritated surface of the stricture. The expedient succeeded perfectly, and the old gentleman had no more constitutional symptoms from the use of the bougie. The same distinguished surgeon urges on the attention of his pupils the necessity of giving no direction to the point of the bougie during its introduction. Gentle force must be used, but the instrument should be allowed to take its own course, otherwise we shall be almost sure of giving it a wrong one and thus penetrate the membrane of the urethra and form a false passage. When the latter accident occurs, which is not very unfrequent in unskilful hands, the best way is to keep the patient quietly in bed, taking nothing but the mildest diluent drinks, and, if there be pain in the parts, to apply some leeches. By this plan the false passage heals, and no ultimate mischief ensues.

---

### 12. M. LISFRANC'S HOSPITAL REPORTS.*

M. Lisfranc continues, with zeal, his hospital reports. Messrs. Geof-

* In La Pitié. Revue Med. Juin, 1826. Recorded by Messrs. Geoffroy and Lambert.

froy and Lambert are the gentlemen appointed to record them, and we hope this plan will soon be followed by all the eminent medical officers of our public establishments. Valuable contributions might now be made to medical science from the naval and military hospitals; and, from the character of those who preside over these departments, we anticipate this event. But to return to M. Lisfranc.

1. *Chloruret of Lime in Burns.* This substance, in rather a dilute solution, has been applied, in La Pitié, to burns of different kinds, and apparently with advantage. The application is sometimes made directly after the accident—sometimes with the precaution of previous emollient cataplasms. The burnt surface is first covered with fine linen slit in various places and spread with cerate. Over this is placed a quantity of lint wet with the solution of chloruret of lime, more or less strong, according to the idiosyncrasy of the patient. The dressings should be frequently moistened with the solution, and never permitted to get dry.

Burns are divided, by M. Lisfranc, into six degrees of intensity: viz. 1mo. Superficial inflammation, without phlyctenæ; 2ndo. Inflammation of the skin with phlyctenæ; 3tio. A portion of the papillary substance of the skin blackened, the vital powers of this portion extinguished, but the alteration not extending deep; 4to. The whole substance of the skin burnt and deprived of life; 5to. All the tissues disorganized, the bones only left untouched; 6to. The soft and hard parts reduced to a state of carbonization.

M. Lisfranc details seven cases of burn, produced in various ways, and by different burning substances, which were treated with success in La Pitié. They were almost all burns of the *third* degree according to the above scale, some of them being only of the second degree of intensity.

Whether this mode of treatment possesses advantages over the cold applications, or the opposite dressings of turpentine, &c. which are in use on this side of the channel, must be decided by clinical experience. We here put our readers in possession of M. Lisfranc's plan, without hazarding any opinion of our own.

2. *Cancer of the Rectum removed by Excision.* This was a formidable operation, and, if the disease was really of a scirrhous nature, the event has been most fortunate indeed, for terrible is the fate of that man who is destined to end his days by cancer of the rectum!

*Case.* Joseph Pontain entered La Pitié on the 9th February, 1826, aged 45 years, of good constitution and sanguineous temperament. He stated that he had laboured under hæmorrhoids for 15 years, and never used any remedies. For five months previously, the pourtour of the anus presented a number of nipple-like eminences very close together, the patient experiencing great pain whenever he went to stool. When any portion of the rectum was everted by straining, it appeared studded with eminences similar to those which were external. When he entered the hospital his general health appeared good, and all the functions carried on with regularity.—On the first examination, M. Lisfranc considered the case as one of piles, and prescribed accordingly; but, a few days afterwards, on introducing the finger into the rectum, he recognized a scirrhus of that gut, occupying about an inch in extent of the mucous membrane. The patient experienced lancinating pains, of an intermitting character in the part, and was told that nothing but an operation would cure him. To this he readily assented, and it was performed in the following manner. The patient being laid on a mattrass, an assistant introduced a square piece of linen into the rectum, with strings attached to the angles. The hollow of this compress was then stuffed with pieces of lint, so as to distend the rectum above the sphincter, and afford a mean of pulling down and inverting the gut; but the manœuvre did not succeed, as the plug came away without everting the rectum. M. Lisfranc himself repeated the experiment, but with similar want of success. The patient was then desired to strain with all his force, while Lisfranc introduced the fore-finger of the left hand and bent it into the form of a hook, by which he was enabled to invert, though imperfectly, the intestine. He now, with a pair of strong crooked scissors, partially detached a portion of the scirrhus from the subjacent structures, which portion, being seized, served as a tractor to draw the rest more into view, when the whole was completely cut away circularly, thus removing full two inches of the mucous membrane, embracing one half of the sphincter ani. Very considerable hæmorrhage followed, which was suffered to continue for ten minutes, during which, more than a pint of blood was lost. The rectum was then plugged, and a T bandage applied. M. Lisfranc and three pupils remained more than an hour with the patient. No bad symptoms followed during this period. About noon, the patient experienced colicky pains and an urgent desire to go to stool. The plug was expelled by these efforts, and about 12 ounces more of blood were lost. The pulse then became small and intermitting, and syncope was threatened. The rectum was again cautiously plugged, taking care not to push the lint through the wound among the coats of the gut. At four o'clock, P. M. the patient complained of chilliness, the face was a little flushed, the pulse rose, and other febrile symptoms set in, but disappeared in the evening. At midnight, the patient made another effort to evacuate the rectum, and again dislodged the wadding, with a considerable quantity of *coagulated* blood. The appearance of the blood induced the pupil of the night to conclude that the hæmorrhage was stopped, and, therefore, he did not renew the plugging. The patient complained of no severe pain, and he passed the remainder of the night comfortably. Next day, the symptoms were favourable—the pain moderate—and little or no fever present. A poultice applied to the anus. Severe abstemiousness. No untoward symptom afterwards occurred—a rectum bougie was regularly introduced to prevent stricture of the gut—the wound cicatrized, and the patient was discharged cured in a short time.

This operation was very fairly managed, and the only doubt in our minds is in respect to the nature of the disease cut away. We know that, in hæmorrhoidal affections, there will often be indurations and even ulcerations in the rectum, which are very far from being of a cancerous nature, and may continue for a long time, especially induration, without much inconvenience. We suspect that the case in question was one of this kind—and that the removal of the hæmorrhoidal tumours would have been sufficient, without resorting to so formidable an operation as that which was here practised. However, the event was fortunate, and the patient has certainly obtained a radical cure.

3. *Morbid Sensibility of the Retina.* M. Lisfranc reports some cases of both deep-seated and superficial inflammation of the eye, where, after repeated depletion by leeches, &c. and the application of blisters, the phlogosis was dispelled, but the morbid sensibility of the retina to light still continued. In such cases, the application of belladonna, after depletion, was of singular efficacy in taking off the nervous irritability of the retina. These cases we need not detail; but only allude to the practice, which we believe is adopted by the best oculists of this country.

---

### 13. TREATMENT OF HERPES ZOSTER.

M. Geoffroy, of La Pitié, has published some cases of this disease, of which we shall take some notice in this place. Our readers are aware that the lunar caustic has been applied to the variolous eruption by some continental practitioners, and this suggested the idea of making the same application to Herpes Zoster.

*Case.* F. Venet, was received into hospital on the 21st of August, being of robust constitution and sanguineous temperament, affected with zona for seven days previously. His tongue was yellow—mouth bitter—pain in the epigastrium—bowels constipated—thirst—anorexia —pulse rather quick and sharp.

The zona extended from the vertebral region to the umbilicus, about three inches in breadth—very red, with a number of small pustules scattered over its surface, similar to those produced by the tartar-emetic ointment. This eruption was very painful, and compared by the patient to what would result from the application of a hot iron. On the 22d August, M. Serres cauterized part of the zona with a stick of caustic dipped in water. This operation produced no pain. *23d.* The patient says he has had no uneasiness in the part cauterized since yesterday. The part not cauterized has been very troublesome. The same operation was therefore extended to the other portions of the zona, and complete success, with a speedy cure, was the result. Two other cases of a similar kind, and with similar terminations are related.

---

14. MR. BELL ON ANEURISM.

If the physician is often embarrassed by anomalies of function and idiosyncrasies of constitution, the surgeon does not always find it plain sailing, even when steering by the compass of minute anatomy. Deviations from natural structure will now and then occur, and tend to perplex the steadiest hand and the sharpest knife.

A large and muscular Negro was admitted into the Middlesex Hospital, with a pulsatory tumour rising out between the heads of the gastrocnemius muscle of the left leg. The artery at the groin was very large, and easily felt; and pressure thereon stopped the pulsation in the tumour. This pulsation could not be subdued, however, by pressure on the middle of the thigh. "For these reasons, Mr. Charles Bell stated, that he should tie the artery lower in the thigh than usual."*

The vessel was easily found under the edge of the sartorius, and, when tied, the pulsation of the artery was distinctly felt against the ligature. An arterial branch had been seen going off from the artery just above where the ligature was applied—and this was purposely cut and secured. The pulsation in the tumour ceased the instant the ligature was drawn, but soon returned. Mr. Bell felt the beating, and observed, that, be the cause what it might, he should do no more. The wound was dressed, and the pulsation still continued. Three days afterwards the pulsation suddenly stopped, and the patient became ill and feverish, having cough, and pains through the body. This remarkable impression on the constitution seemed coincident with the cessation of beating in the tumour. In two days after this the patient gradually sunk and died.

*Dissection.* The whole of the sartorius muscle was affected with inflammation of an erysipelatous character, which had spread along the course of its sheath. Just below the origin of the profunda, the femoral artery divided into two nearly equal branches, which ran parallel to each other till they arrived at the spot where the artery perforates the tendon of the triceps muscle, and here they united again. The ligature had been placed on the more superficial vessel, a little above this union.

In a subsequent clinical lecture, Mr. Bell stated his opinion, that the unfavourable termination was owing to the peculiar condition of the sartorius muscle, and did not arise from the constitution sympathising with the condition of the tumour. We think there can be no doubt that the constitution sympathised with the erysipelatous inflammation of the muscle and its sheath—but we think, also, that the stoppage of circulation in the tumour, and probably in the whole limb afterwards, was the main or efficient cause of death.

Mr. Bell remarks:—"The surprise is now, not that the tumour

* We wish Mr. Bell had stated *his reasons* for this determination. Did he foresee a bifurcation of the artery in the middle of the thigh? We think he did not, in consequence of an expression which he afterwards used, when he found the pulsation continue notwithstanding the application of the ligature.—*Rev.*

should have continued to beat, *but how it should have ceased to beat by a ligature being put only on one of the arteries, either of which was fully sufficient for the circulation of the limb.*" We cannot discover much cause for surprise at this phenomenon. One half of the arterial current was suddenly cut off by the ligature—and was it unreasonable to expect that a temporary failure of perceptible pulsation would be the result, till an increased current was directed along the other branch of the vessel?—One of these branches might be quite "sufficient for the *circulation* of the limb," in its then calibre and current, though it might fail to produce the phenomenon of *pulsation*, till both were augmented.

Could it have been anticipated that a bifurcation of the artery existed in the middle of the thigh, from the experiment made by pressure, as above stated, would it not have been more feasible to tie the artery at or near that part where pressure actually stopped the pulsation, than to tie it "lower in the thigh than usual," with the hope of being below any reunion of the vessels, if such had taken place? This question can doubtless be solved by the ingenious surgeon who was concerned in the above case.

---

### 15. INVERTED TOE-NAIL.*

This is a very distressing complaint, and the radical cure, as practised by Dupuytren, is a very severe operation. We believe the chiropodists of this metropolis, endeavour to remedy the evil by scraping the nail very thin, at a little distance from where it turns down into the flesh, or rather where the fungous granulations turn up over the nail. When it is rendered thin and flexible, the edge of the nail turns up gradually, and, by destroying the granulations with caustic, a cure is effected. There are two distinct varieties, however, of this complaint—one, in which the root of the nail and the contiguous integuments are free from disease—one side or angle only of the nail being buried in the integuments. In the other variety, the nail is surrounded entirely by fungous granulations, and its root is diseased. On this distinction are founded two distinct modes of cure. In the former case, the removal of that portion of nail which is covered with granulations, will be sufficient—in the *latter*, the whole nail, together with its matrix, must be removed.

In the first variety, M. Dupuytren takes a pair of strong straight scissors, one blade of which is very sharp and thin. The point of this blade he introduces beneath the nail, and, by a rapid and adroit movement, slits it up the middle, and thus divides it into two halves. Then, seizing the anterior portion of which ever half is intended to be removed, with strong forceps, he turns it back, and by the aid of the scalpel, dissects it entirely off. The same is done with the other half, should there be disease (as is sometimes the case) on both sides of the toe. After this operation, patients are frequently turned out of the Hôtel Dieu cured, in eight days.

---

* De la Cure radicale de l'Ongle Incarné, par le Procedé de M. Dupuytren. *Archives, Juillet*, 1826.

*Second Method.* In this case the surgeon seizes the extremity of the great toe with the finger and thumb of his left hand, while, with a convex bistoury, he makes a semicircular incision a little behind the root of the nail, from one side to the other. He then seizes the extremity of the nail with strong forceps, and, reversing it backwards, removes it and the portion of soft parts anterior to the semicircular incision. By this operation, the root of the nail and its matrix are removed, and there is no danger of reproduction of the disease. The part is to be dressed with cerate after the operation, and if the pain be considerable, some opiate may be put in the dressings, and a cataplasm applied. If the patient keep quiet, a cicatrix will be formed in eight or ten days.

---

### 16. DR. GRANVILLE ON GASTROTOMY.

The attempts—one of them successful—which have been made in this country by Mr. Lizars, to remove ovarian disease by an operation, will probably induce many surgeons to hazard the *anceps remedium* of gastrotomy, rather than see their patients die a lingering death. In our review of Mr. Lizars' work, we gave it as our opinion that—"when a diseased ovarium arrives at that size which requires an operation, there must be such adhesions to the neighbouring parts, as to render success, after an operation, next to a miracle.* Let us see whether this prognostication be confirmed or refuted by Dr. Granville's operation, recorded in the August number of the Medical and Physical Journal. No history or description of the disease, prior to the operation, is given by Dr. Granville. The patient was prepared for gastrotomy by a fortnight's repose, and three days' use of prussic acid—"a passive temperament, and almost total absence of irritability," being considered very favourable circumstances in her constitution. An incision being made through the integuments of the abdomen, the intestines and part of the curvature presented themselves, "in rather a vascular condition;" when Dr. G. passed his hand into the abdominal cavity, and ascertained that—"although the tumours felt loose, when examined externally, they were, in fact, adhering in various directions by strong bands." After some farther examination, in which Mr. Brodie and Mr. Keate satisfied themselves of the above-mentioned state of the case, the enterprise was given over—the wound secured by hare-lip pins—and the patient put to bed. Fortunately this attempt was productive of no fatal consequences, as the woman rapidly recovered, without any bad symptoms.

We leave our readers to make their own reflexions on this case. As far as it goes, it confirms our prophesy—while it adds another to several instances now on record, "of the impunity with which the cavity of the abdomen may be laid open." We would not, however, advise the enterprising surgeon to place too much confidence in this impunity—or to expect that gastrotomy will always, or even generally,

* New Series, vol. 3, p. 336. October, 1825.

be attended with so little inconvenience, in the event of its being deemed imprudent to proceed farther in the operation.

---

### 17. REMARKABLE CEREBRAL AFFECTION.

Organic diseases of the brain are amongst the most terrible afflictions to which human nature is liable. When the centre of sensation and the seat of thought becomes altered in structure, the sufferings of the individual call loudly for the grave, as the only refuge from a penal existence of indescribable wretchedness! Such was the case of an unfortunate female in St. Bartholomew's Hospital, under Dr. Latham. For eight months previous to her entry, (22d Nov. 1825) she had suffered pain in her head, which was suspected to be of a syphilitic nature, and was treated by various means, without relief. She had even been profusely salivated. She exhibited a peculiar sense of terror and alarm in her countenance, with a trembling gait, which were found to be owing to a continual effort to give steadiness to the head. She soon became permanently confined to bed, and unable to lift her head from her pillow during the greater part of the day and night. Her pulse was so feeble as to contra-indicate venesection, though the throbbing of the temples called for leeches to the temples, and cupping. The relief was merely transitory. Mercury was again tried, but she grew worse under its administration. Oil of turpentine, quinine, conium, belladonna, were useless—some of them detrimental. And now a large abscess formed in the axilla, and gave vent to an enormous quantity of pus, the discharge continuing for three weeks, at the rate of a pint per diem. During the continuance of this discharge the agonizing head-ache entirely ceased—the patient left her bed—walked about the ward—and described herself as free from all ailment, exulting in the certainty of restoration to health. Even her medical attendants naturally enough concluded that the previous cerebral pains did not depend on organic derangement of the brain. But as the purulent discharge abated, the former symptoms returned. A large caustic issue was, therefore, established on the inside of the humerus, and a copious discharge elicited. But this was of no avail. Mercury was again given, and salivation excited, without benefit. Death, at length, put a period to the most dreadful state of suffering that can be conceived.

On dissection, the brain and its membranes were found more vascular than natural, and some effusion under the arachnoid and in the lateral ventricles. At the base of the cerebellum, and growing from both lobes, was a tumour which descended beneath the dura mater into the spinal canal reaching as low as the origin of the sixth pair of cervical nerves. It was of the consistence of fœtal brain, and the contiguous portions of cerebellum were softened. Some blood was extravasated upon the pia mater of the spinal marrow. No other disease was found.

*Remarks.*—The principal anomaly in this case, was the cessation of the pain and other symptoms, for a time, while the organic disease must, of course, have existed. Unfortunately for medical science this kind of anomaly is of no infrequent occurrence. We see the functions of organs apparently little, if at all, deranged, while their structure is irrecoverably altered. This we observe in the brain, lungs, stomach, heart, and other parts; and the recollection of this should always keep the practitioner on his guard in respect to prognosis as well as diagnosis. There is another peculiarity in Dr. Latham's case—the intensity of the sufferings, as disproportioned to the extent of disorganization found on dissection. How often do we find an immense mass of disease in the brain, without any thing like a corresponding degree of suffering during life. These are some of the obstacles to *certainty* in medical science. They may, at least, teach us *humility* in the exercise of our profession. —*Med. and Phys. Journ*, *No.* 1, *N. S.*

---

### 18. DEATH FROM AMPUTATION OF A TOE.*

The danger of an operation is not always in proportion to the magnitude thereof; and death has sometimes been the consequence of amputation of a crooked finger or toe. In the following case there was something very particular, which is worthy of record.

*Case* 1. Henrias, aged 19 years, perceived, about six months before he entered the Hospital of the Faculty, a slight swelling of the second toe of the right foot. This gradually increased—broke—and an ulcer of bad character formed. When admitted, 4th of May, 1826, it was found that the first phalanx of the toe and the joint were diseased, and amputation was performed, without difficulty, in the metatarsal bone. Union by the first intention was attempted. On the 1st, 2d, and 3d days after the operation, there was some fever. On the 4th and 5th there appeared swelling and inflammation on the back of the foot. This ended in suppuration; and erysipelatous inflammation kept creeping upwards, with fever. On the 9th day the skin was jaundiced—but the constitutional symptoms were not severe. An inflammation was observed on the back of the left hand, where fluctuation was felt, and in another day or two a large collection of pus, of very bad quality, was discharged by an opening made with the bistoury. From the foot, an immense quantity of purulent matter was draining, mixed with blood, and of an insupportable fetor. There was now delirium at night—pulse small and irregular—features shrunk—respiration laborious. He died on the 11th day from the operation.

*Dissection.* Passing over a minute detail of the appearances in the foot, we shall only remark that the veins leading from the wound were red, thickened, and filled with pus, which was also found in the saphena vein, and in the veins of the left upper extremity, leading from the abscess in the hand. In the head there was no morbid appearance. In the left lung was found a considerable number of small abscesses,

---

* Hôpital de Perfectionnement. M. Velpeau. *Archives*, Juillet.

varying in size from a pea to that of a nut, and filled with pus. In the left cavity of the pleura there were about three ounces of a yellowish fluid. The ventricles of the heart were empty—but in the auricles some purulent matter was found, mixed with fibrinous concretions. In the abdomen, there was a small ulcer at the ileo-cæcal valve, apparently of some standing, and the glands of Peyer and Brunner were becoming somewhat prominent; but there was no redness or disease of the mucous membrane of the intestines. There was an astonishing vacuity of the blood-vessels generally. Not a drop of blood was seen in the various branches of the vena portæ, and the trunk of the vena cava was lubricated with a puriform fluid similar to that which was found in several other parts of the body. The icteritious colour pervaded not only the surface, but every organ and structure, even the bones, cartilages, brain, muscles, &c. &c.

*Remarks.* This case is brought forward (with many others) not on account of the fatal termination of an operation, but as a specimen of the pathology of the fluids—or in other words, of the humoral pathology, and the important part which it plays in diseases. Here the whole blood of the body, with the exception of about four ounces, was in a state of decomposition, and every tissue and structure imbued, as it were, with this morbid circulating mass. Our author asks, "can we deny that the alteration of the fluids was the source of the morbid phenomena and the cause of death in this case?" The *latter* part it would be difficult to deny. But a question might still remain, was not this degeneracy of the fluids the *consequence* rather than the *cause* of the erysipelatous inflammation in the foot, the phlebitis, and the admixture of purulent matter with the blood, in the first instance? These questions we leave for the physiologist and pathologist to solve. The case above-related is one which bears strongly in favour of the humoral pathology, so much neglected in modern times.

*Case* 2. Flevenaux, aged 62 years, entered the hospital on the 8th May, 1826. He had enjoyed good health till two years previously, when he began to feel pain in his bowels, and especially shooting pains while making water. From this period his digestion became much deranged, the pain in making water continuing, but the abdominal uneasiness ceasing. Œdematous swellings had commenced for some time, and at the time he entered the hospital, the lower extremities were infiltrated throughout. On examination, the abdomen was found to be distended by a tumour which occupied the upper half, and almost the whole of the left side of that region. Skin pale and blanched, as in anemia—pulse and tongue natural, as was the respiration—alvine secretions scanty—the principal complaint of the patient was want of sleep and pain in making water. Lavements—diluent drink—tepid baths, were at first prescribed—then a grain of opium every night, which procured some sleep. The dropsical effusions gained ground in spite of diuretics. Ascites appeared to obtain—debility increased—and death took place on the 6th June.

*Dissection.* The head and spine were not examined. The lungs were found to be studded with tubercles of various sizes, containing a substance resembling brain—some of them crude, and others softening down into a fluid state. In the abdomen, there was no effusion—the peritoneum was healthy—the cæcum, colon, and a great part of the small intestines were pressed forward by a tumour the size of a boy's head of ten years of age. The liver was three times its natural size—the other viscera sound. The tumour above-mentioned was formed by a diseased kidney of that side, transmuted into a mass of cerebriform substance, hollowed out into a number of pouches or sacs. In the pelvis of the kidney, and branching into several of the sacs, was a large clot, two inches in diameter, of a pale yellowish substance, unattached to any part, and somewhat resembling the female roe of fish when boiled. The liver presented no mark of inflammation on its surface—it occupied the whole of the right, and most of the left hypochondrium, descending as far as the navel, but without adhesion to any surrounding viscus. The whole of the organ was studded with tumours of cerebriform substance, varying extremely in size, from a hemp-seed to a swan's egg. These tumours were sometimes like tubercles, and contained in cysts—sometimes in a diffused form, infiltrated, as it were, among the parenchyma of the organ. It was all soft. The parenchyma itself of the liver did not appear altered in structure, except from admixture of this cerebriform matter, which pervaded the whole viscus.

There was nothing very particular in the veins of the lower extremities or pelvis. The cava inferior and superior, together with the auricles, contained fibrinous clots, apparently of long standing, and when broken down between the fingers, resembling the cerebriform matter which was found in other parts of the body. The blood itself in the vessels appeared very different from that which is usually seen—but which M. Velpeau declines characterizing. The parietes of the veins were every where sound.

*Remarks.* It is evident that, in the above case, there was one general constitutional disposition to the formation of this cerebriform matter; but, in what way this morbid tendency was connected with, or dependent on, a pathological condition of the circulating fluids, is more than we can clearly comprehend. It is, however, much more likely to depend on a vitiated state of the fluids than of the solids; but is not this degeneracy of the fluids dependent, originally, on some derangement of *function* in the vital viscera, and especially of the organs of supply—the stomach and the whole digestive apparatus? This appears to us the most probable theory—and moreover the theory which is likely to lead to the best practice, preventive or curative.

---

### 19. NITROUS ACID AND OPIUM IN DYSENTERY, &C.

Mr. Hope, of Chatham, has published (Ed. Journ. July, 1826) some observations on this combination. More than twenty years ago, he was led to its use by accident, and afterwards prescribed it on purpose. Since that period, he has continued the remedy, "with unvaried success," in all cases in private practice. He also experimented on five invalids from tropical climates labouring under chronic bowel complaints, to such an extent, that the medical officer in charge of them considered them as lost cases, and the men themselves had taken the sacrament and were in expectation of death. Three out of the five were cured. In the year 1819, twenty-six men were invalided for dysentery from the West Indies. Fifteen were placed in one ward, and eleven in another. The former were treated with the remedy in question—the latter in the usual manner. Out of the fifteen so treated twelve recovered, and three died. Of the eleven, three recovered and eight died. In the year 1821, our author was surgeon of the Ganymede, where many cases of cholera and diarrhœa occurred, all of which were successfully treated by the same remedy. Afterwards, in the Dolphin, no less than 264 cases of colic, dysentery, and cholera occurred. Of these not one died, having been treated by the remedy in question; the form of which was as follows:—℞. *Acidi nitros.* ʒj. —*mist. camph.* ℥viij. *tinct. opii*, gt. 40—*misce. capiat quartam partem tertiis vel quartis horis.* In chronic dysentery the dose of two ounces three times a day, of the mixture, was sufficient. The remedy is grateful to the taste, abates thirst, removes the intensity of pain, and procures relief of the dysenteric symptoms. The nitric acid was tried, but did not prove effectual like the nitrous.

The remedy above-mentioned has been used in India for twenty or thirty years past—and often with very good effects. But we apprehend that it is not to be relied on in acute cases in hot climates. It is in the chronic bowel complaints succeeding acute, that we have found it beneficial. The acid improves the tone of the stomach and bowels, while the opiate lessens their irritability and restrains the mucous discharges. This, we venture to presume, is the rationale of the effects produced by the combination in question, and the only expectations which can be entertained with safety. Nevertheless we promulgate Mr. Hope's sentiments, and wish every success to subsequent trials of the medicine.

In the same number of our respected cotemporary, Dr. Burke, has presented us with a paper on the good effects of a mixture of acetate of lead and tincture of opium in an epidemic dysentery which prevailed among the lower Irish of the city of Dublin, in the summer of 1825. The formula used (making allowance for age, &c.) was as follows:—℞. *Acetat. plumbi*, gr. iv. *tinct. opii*, ʒij. *aquæ distillat.* ℥ij. *ft. solutio. capiat æger* ʒss. 4$^{ta}$ *quaque hora:* Now, when we consider that each dose of this mixture contained thirty minims, or about fifty drops of laudanum, and that every fourth hour, we can hardly be surprised that "the symptoms were mitigated—the tenesmus abated—and the tormina ceased"—or that a patient should describe herself, after the second dose, "as if she were in Heaven." In many of the bowel complaints of summer and autumn in this country, opium will give relief, and indeed effect a cure. Not that we maintain that the simple exhibition of this medicine is as good as its combination with other agents, as antimony, calomel, &c. On the contrary, we are convinced that the latter is infinitely preferable to the former. In respect to the acetate of lead conjoined with laudanum, we have only to say that it has often been employed, and found useful in weakly habits, and where but little inflammatory action obtained. In the acute dysenteries of warm climates it would not answer, but would be almost certain of doing mischief.

---

### 20. PARACENTESIS THORACIS.*

In our ninth Number, we introduced a successful case of this operation, performed by Mr. Jowett, at Nottingham. We now present our readers with another instance which, though not so fortunate, is yet interesting. It occurred in a public institution, and consequently where the particulars have been fully authenticated.

*Case.* Serry, a Negro servant, was seized with pneumonia of the *right* lung, for which he was bled, blistered, cupped, &c. with apparent removal of the inflammation. The cough, however, continued, and the treatment was neglected. In two months afterwards, he was again attacked with pneumonia of the same lung, of considerable intensity. It was treated vigorously; but as Serry had a bad place to sleep in, he became affected with pleurisy of the *left* side. At this time, examination by percussion shewed the lower portion of the left side devoid of sound, while, above the fourth rib, it was unnaturally resonant. By the stethoscope, the total absence of respiration in the left lung was determined, not only in front, but in the axilla and between the scapula and spine, except very high up where it was indistinct. "These signs were satisfactory evidence that an effusion, both of liquid and air, had already taken place into the sac of the left pleura." Dr. Jackson was convinced, from the absence of any loud sibilant rattle, that the air was not derived from a communication with the lungs, and that the effusion did not consist of pus, proceeding from an abscess that had ruptured in the lungs, but was serum, the result of acute inflammation of the pleura. It is unnecessary to detail the treatment pursued. In the course of a fortnight, all the acute symptoms had disappeared—a copious mucous expectoration was established—the breathing was unembarrassed—the skin soft and moist—and the general feelings comfortable. Exploration of the chest presented no alteration in the left, except that the metallic tinkling had disappeared. In the right lung, a mucous and slight crepitous rattle still existed, and the cough continued. In another fortnight, œdema occurred, together with hurried breathing and dyspnœa on ascending the stairs. On examination with the stethoscope, it was

* Dr. Samuel Jackson. Philadelphia Almshouse Infirmary. Philadelphia Journal of the Medical Sciences, No. 1, New Series.

evident that pneumo-thorax and a serous effusion existed in the left side, which was found to be an inch and a half larger than the right: succussion was also performed, when the splash of a liquid in the chest was distinctly heard.

*Operation.* This was performed by Dr. Gibson, in the presence of a large class of pupils, at the Infirmary. The incision was made between the sixth and seventh ribs, at the point of their greatest projection. Half a gallon of a clear serous fluid, mixed with some flocculi of coagulated fibrin, was drawn off. The only inconvenience experienced was a severe fit of coughing, induced by the introduction of a gum-elastic catheter. No sooner was the effusion removed than pain was complained of in the pleura, which required two bleedings the same day. Next morning, the symptoms were relieved; but cupping and blisters were necessary. From this time, the pleuritic symptoms gradually declined, and the patient could soon lie on either side. In three weeks, the whole of the left side was resonant, but no respiratory murmur could be heard in any part of the lung of that side. From this it was concluded that the lung had not expanded, but remained compressed by the air contained in the pleura, or from a change in its structure.

Exploration of the right side gave reason to fear that tubercles were developing there, and that phthisis would be the result. The antimonial ointment was rubbed on both sides of the chest, and strict regimen enjoined. From this time, the patient complained of pain in both sides of the chest, and, in ten days more, the symptoms were much aggravated—the pain being permanent and acute—the respiration embarrassed. The left side returned a dull sound below the fourth rib—hollow above that point. The right side was less resonant than natural in its lower portion, where acute pain was felt. On succussion, the splash of a fluid was again discernible. The symptoms of phthisis now became developed. On the 10th February (the operation was performed on the 15th December) the wound burst open, with a loud explosion, during a fit of coughing, and gave vent to a considerable discharge of clear serum containing coagulated flocculi, with relief to the feelings of oppression. The patient died of decided phthisis on the 11th March, upwards of three months after the operation of paracentesis thoracis.

*Examination.* On raising the sternum, the right lung did not collapse, the left side presented a cavity. The right pleura was inflamed, the lung was solid, containing tubercles in various stages—some of them broken down and evacuated—two cavities, whence the pus had issued, and where pectoriloquism had been heard. The left lung was compressed to one fourth of its original volume, and solidified, but not otherwise changed in structure. The pleura pulmonalis and costalis was covered with a thick coating of coagulated fibrin.

From the history and dissection, it was evident that death was occasioned by phthisis of the right lung, and not by the pleuritic affection or the operation in the other side. Had this alone existed, it is probable that life might have been saved.

We congratulate our American brethren on their zeal and proficiency in auscultation. They are certainly outstripping, in this respect, the generality of practitioners in the metropolis of Great Britain, where this interesting study is far less cultivated than it deserves to be, owing to the supineness of the higher orders of the profession here. This is an unpalatable potation; but bitter medicines are more wholesome than sweet.

### 21. MASKED CEREBRAL AFFECTION.

Dr. Chambers has reported, in our respected cotemporary, the case of a woman, Mrs. Miles, whom we attended previously to her admission into St. George's Hospital. When she first applied to us, she complained of discharges of blood from the lungs by coughing. This was stopped by a few doses of super-acetate of lead and opium. She next complained of sickness of stomach whenever she took food, together with pain in the occipital region of the head. Blisters were applied to both parts, and medicines were given to quiet the gastric irritability, but without success. The patient having but bad accomodation in the Little Theatre of the Haymarket, we sent her to St. George's Hospital. There the sickness at stomach and pain of the head were treated with the same want of success as before. There was no tenderness of the epigastrium or abdomen—pulse from 80 to 90—tongue clean—skin cool—bowels constipated. The intellectual functions were never disturbed. Among other remedial agents, she was placed under the full influence of mercury—an issue was inserted in the back of the head—blisters were applied to the epigastrium—opium, subnitrate of bismuth, &c. were given internally—but all to no purpose. At length she died exhausted by the progressive increase of the symptoms. Some curious speculations were hazarded as to the nature of the disease, and one gentleman, who could see deeper than the rest, considered it as a tuberculation of the peritoneum. It was *not* Dr. Chambers.

On dissection, no diseased appearance was found in the stomach or bowels. In the centre of the posterior lobe of the right hemisphere of the brain was a small tumour, the size of a hazel nut, somewhat softer than the contiguous brain. A similar mass was found in the posterior lobe of the left hemisphere. The left lobe of the cerebellum was almost entirely destroyed by the suppurative softening of a similar tumour occupying its interior. The surrounding cerebellic substance was softened, and there were three ounces of water in the ventricles.

This case illustrates well the sympathetic effects of cerebral disease on the stomach. It was of the latter organ that the patient chiefly complained. With all this organic disease of brain, the intellect was unimpaired to the last.

22. BRONCHOCELE—LIGATURE OF THE THYROID ARTERIES.*

We are of opinion that this disease is more common than it was 30 years ago, in this country. However this may be, English women (for it is far more common in the female than in the male) cannot bear with so little impunity as in Switzerland and other goitrous countries, considerable enlargements of the thyroid gland. We know two ladies in this metropolis who are suffering under distressing dyspnœa, in consequence of a bronchocele, which would scarcely be called one among the Alps. One of them is deriving much relief from iodine, while in the other it produces so much distress, when given internally, that she cannot continue it. We recommend to the attention of our brethren the observations of M. Lisfranc, in this number, on leeching in white swellings and mammary enlargements. A small number of leeches will produce excitement in bronchocele, and thus increase the disease; while a large number at once will have a very contrary effect. This we have seen take place unequivocally, of late, in a case of goitre. In many instances all remedies will fail, and then, perhaps, the experiment of Mr. Earle may be worthy of trial.

*Case.* J. Larking, aged about 18 years, became affected with bronchocele when she was only thirteen, and the swelling, excepting for two years, when she menstruated regularly, has been progressively increasing. In 1822, she was in Bartholomew's Hospital, under Mr. Earle, when the swelling was large, and much inconvenience experienced in breathing and swallowing. Her general health was bad, and attention was paid to this by diet, and by improving the secretions. Under this plan she got a little better, and then left the hospital. In 1823, she returned from the country, the bronchocele having increased as her general health amended. It now threatened suffocation at times, she was wholly incapable of swallowing any solid food—pulse 120—bowels costive—catamenia stopped for five months past—cough and pain in the chest had returned, with constant head-ache and drowsiness. The superior thyroid arteries were much enlarged, especially that on the right side, which led to the idea that its coats were diseased. Leeches—evaporating lotions—the blue pill—and the tincture of iodine were employed—but the latter, producing nausea, was soon discontinued. In a fortnight, it was evident that the swelling had increased, and her breathing was become so extremely laborious that it was clear she could not long survive, if some relief was not obtained. Under these circumstances Mr. Earle threw a ligature round the right superior thyroid artery. The vessel was found to be healthy, but nearly as large as the carotid. The most acute pain in the head followed the tightening of the ligature. The pulsation in the tracheal side of the artery diminished materially, but did not entirely subside. About half an hour after the

* Case of Bronchocele, in which the thyroid arteries were tied. By H. Earle, Esq. of Bartholomew's Hospital. Lond. Med. Journ. September, 1826.

operation, the head-ache continuing, 20 ounces of blood were abstracted, with some relief. Cold applications were made to the tumour, and the head kept elevated. In the evening the carotids were beating with more violence than before the operation. Saline purgatives—digitalis. She passed a bad night; and next day her pulse was rapid, tongue furred, drowsiness amounting to coma. The bowels were opened with calomel and jalap, and 20 leeches were applied to the temples, which afforded great relief. In the evening all the bad symptoms were abated, and the breathing was much easier. She continued to improve. Three days afterwards, the tumour was found to be considerably smaller—respiration and deglutition performed with comparative facility. The pulsation was much reduced—cough nearly gone. In five days more the neck was measured, and the tumour was found still farther diminished. The portion of the artery between the ligature and the carotid had ceased to beat. She could breathe and swallow easier than during the last two years. The ligature came away. She was sent into the country, under the promise of returning, should the tumour increase. In a fortnight she came back, the tumour remaining stationary, but the left thyroid increasing in pulsatory force. This artery was secured also, being healthy in structure, and about the size of the radial artery. Leeches had been previously applied, and her system depleted by saline purgatives. No alarming symptoms followed the ligature. The subsequent diminution of the tumour was not near so great or so rapid as after the first operation. The menses reappeared, after an absence of seven months. In about three weeks she finally left the hospital, having no cough, no impediment in swallowing, and her breathing free. The tumour was evidently smaller and softer, and appeared more divided in texture. In 1824, Mr. Earle heard from the patient, when the tumour was reported to be slowly decreasing, and the patient's health much restored.

This is certainly a gratifying case, as it appeared at one time very threatening. It is curious that the cutting off one artery out of four should have had such an effect, as was the case after the first operation.

---

### 23. CANCRUM ORIS INFANTIUM.*

This curious and dangerous disease is described in Pearson's Surgery, and copied into Cooper's Surgical Dictionary, under the head of cancrum oris, and characterised as a deep, foul, irregular, fetid ulcer, with jagged edges, and appearing upon the inside of the lips and cheeks of children, between the age of 18 months and six or seven years.

Ever since the establishment of the Children's Asylum in Philadelphia, the institution has been annually visited by this distressing scourge, especially in the winter season, its destructive ravages being marked by

---

* Description of the Gangrenous Ulcer of the Mouths of Children. By B. H. Coates, M.D. one of the Physicians to the Philadelphia Children's Asylum, &c.—*North American Med. and Surg. Journ. July*, 1826.

an insidious approach and too often uncontrollable progress. But few and scattered notices of this complaint can be found in practical writers, and we do not mean to follow Dr. Coates in his imitation of some modern authors, who cannot describe a single disease or scarcely a symptom of disease, without first piling and compiling together all that has ever been said or thought of the same thing since the flood of Noah. This practice may be the revival of learning, but it will prove the decay of knowledge. People are now afraid to buy, and will not read, books that are composed of the rakings of a musty library, with here and there a solitary fact or original thought. These reflections are not levelled at Dr. Coates, but have been merely excited by his paper. *He* has been very moderate in his researches, and only occupies six pages and a half with these fashionable specimens of erudition. Some of our modern book-worms would have spun them out into ten times that space.

*Locality of the Disease.* The Asylum is built on an alluvial soil, and though well situated in itself, has a marshy ground on its south side.

*Description of the Disease.* In the majority of cases, the ulcer begins immediately at the edges of the gums, in contact with the necks of the teeth.

"A separation is found here; which exhibits a slight loss of substance at the extreme edge of the gums, and, as far as I have observed, a whitishness of the diseased surface. In some instances, though not very frequently, this is preceded by a slight swelling and redness. In this state, the disease may continue for a long time; and I have reason to believe, that patients have remained thus affected, during the whole period of three months, for which I attended the Asylum. At one time, when the disease was at its height, threatening several patients with destruction, I found upwards of 70 children, out of a population amounting to about 240, more or less affected with these ulcerations. No remarkable change is at this stage observable in the functions of the little sufferer; except a general air of languor and weakness. The appetite and the muscular activity continue, but are somewhat reduced; not sufficiently, however, to disable the child from attending school, taking the air, or continuing his ordinary practices. In this state, no symptoms of irritation have been at all discovered. The skin is cool during the day, no pain is complained of; and no account has ever been given me of any nocturnal paroxysm of fever. It would appear to be purely a state of asthenia. We are, however, by no means certain, that there was no concealed irritation in the system. We were, of necessity, obliged to depend, in a great measure, upon the reports of nurses, and other females: and these were liable to overlook, or mistake for mere weakness, the signs of an obscure disease. In this manner, commencing cases were frequently not discovered, and nothing was done, till the affection had made further progress; and this continued until the ascertained existence of the epidemic in the house, combined with the recollection of its former ravages, had excited an alarm, which led to the inspection of the mouths of all the children in the institution.

"The disease, in this form, must be within the curative powers of nature; as, if this were not the case, we should hear of more numerous unfavourable terminations. It has seldom, however, if at all, been within my power to witness this tendency; and, when not controlled by a particular treatment, the cases have almost always either remained stationary, or increased in severity. Its first progress is, most generally, by extending to the edges of the gums round other teeth; frequently affecting a large portion of the dental arches. A very early progress is, however, mostly effected, down the length of the tooth, in the direction of the socket; and, in this way, the disease commits great and unsuspected ravages. When it reaches the edges of the bony socket, the tooth begins to be loose, and when drawn, exhibits portions of the fang, including parts which had been contained within the alveolus, entirely denuded of their periosteum. Indeed, from observation, I should say, that the latter membrane was the part, which was the most peculiarly liable to injury and death from this disease; and it is by no means clear, to my apprehension, that this is not frequently the commencement of the complaint. The injury generally proceeds with augmenting rapidity; especially when it has affected the deeper parts; and it is while in the act of rapidly spreading, that it occasions gangrene.

"In the production of gangrenous sloughs, it much resembles the descriptions usually given of sloughing ulcers. A portion of the parts immediately subjacent to the ulcer loses its life; this rapidly separates; and, before or after a complete removal, a fresh slough is formed in the same manner. The sloughs are generally black, with ash-coloured edges. I have not been able to discern a change of colour, the production of vesicles, or any material tumefaction, as antecedent to the gangrene. There is, generally, by this time, an increased heat in the parts; with the sensation termed "calor mordens." The discharge now, for the first time, becomes acrimonious; giving pain when it comes in contact with cuts in the finger; and excoriations are produced on all parts in contact with the sloughing ulcerations; as the lips, the cheeks, the tongue, and the adjoining surface of the part where the ulcer is situated.

"As soon as the external gangrene has reached the level of the edge of the bony socket, and frequently much sooner, the adjacent portion of the latter is found deprived of its life; forming a necrosis. The death of the periosteum in the socket, at least that of the fang of the tooth, precedes, by some interval of time, that of any portion of the bone itself.

"When gangrene is formed, a fever of irritation is generally developed. In regard to the time at which this takes place, there is a great diversity in different constitutions. It has appeared to me to depend, principally, upon the inflammation of the mouth, which is secondary to the original disease, and, in most cases, to arise from the acrimony of the discharge. It is aggravated by loss of rest, want of nourishment, and, probably, by putrid matter finding its way into the stomach. To the latter cause I also refer a diarrhœa, which almost uniformly comes on, towards the close.

"There are accounts of a similar disease having begun on the inside of the cheeks. I have, however, never seen a well-marked instance of this; the cases which were supposed to be such having, in every instance, been also found to exhibit ulcerations at the edges of the gums. That the disease spreads from the gums to the cheek, is a fact which I have often seen exemplified. It is, indeed, the most usual termination of bad cases. After producing gangrene and necrosis in the gums and alveoli, and after the discharge becomes, as above stated, acrimonious, a gangrenous spot is not unfrequently found about the opening of the Stenonian duct, on the inside of the upper or lower lip, opposite the incisors, in some other part of the inside of the lip or cheek, or in more than one of these situations at the same time. Whether this be owing to excoriation from the discharge, or to some other cause, I cannot say; it has, however, in every instance which I have seen sufficiently early to witness its rise, been subsequent to the symptoms previously described.

"When the gangrene reaches the cheek or lip, however, very active inflammatory symptoms are uniformly developed. In the cellular substance of these parts, they assume the well known characters which have been attributed to the *phlegmonous* species. We have a great thickening, forming, in the cheek, a large, rounded, prominent tumour, with great heat and pain. Sometimes redness is perceived externally; but, more frequently, the great distention of the skin of the cheek seems to empty the cutaneous vessels; giving to the part a smooth, polished, dense, white appearance, very much resembling the effect of a violent salivation. I have no doubt that this is the tumour described by Poupart, and alluded to in an earlier part of this paper. Great thickness and hardness have always occurred, in the other situations where this gangrene has approached the external cellular masses of the face; in the lip, however, they are less remarkable, perhaps from the smaller amount of cellular matter. After reaching this stage, a black spot is frequently seen on the outer surface of the swelling. This spreads rapidly; and has always been, in my own experience, the immediate harbinger of death. It is proper to state, however, that I have heard it said, that cases had recovered in this city, in which the gangrene had produced a hole through the cheek. Under what physician's care this occurred, I have never learned.

"In two cases it commenced in the fauces; and was marked by the same unsuspected progress. In one of these, the little patient was remarked to be languid, but had no positive external marks of disease. The mouth was examined, and found healthy; but no suspicion of the real situation of the disease was entertained, till after three or four days more, when he complained of a slight sore throat. A large gangrene of the tonsils, half-arches and pharynx, was now found; and the event need hardly be told.

"The closing stage of this affection is marked by large gangrenous patches in the gums; deep fissures between these and the teeth; the latter loose, or falling out; large pieces of the alveolar processes, often containing the roots of several teeth, in a state of entire necrosis; the whole lining membrane of the mouth suffering a violent excoriation; the whole adjacent external cellular substance, hard and swelled; large gangrenous spots in the inside of the cheek or lips, occasionally extending quite through to the outer surface; a total incapability to sleep, or to take the least food; fever; a swelled abdomen, and diarrhœa". 15.

Our author had an opportunity of examining only one child who died of this disease, and dissection threw no light on the malady.

*Pathology.* Although, in some cases, there was apparently no premonitory disorder of the system, yet, in many others, the ulceration followed a common remittent or intermittent fever—"insomuch that, at one time, whenever a child was brought to the nursery for fever, it was expected, as a matter of course, that his mouth would become sore." We dare not follow Dr. Coates in his pathological speculations, but refer to his paper those who are anxious on that point.

Our author tried a variety of remedies with little or no success, for a time—at length he was more fortunate.

"The remedy which beyond all comparison succeeded best, was sulphate of copper. The usefulness of this substance, though known at Salem, New Jersey, was discovered, at the Asylum, by the mistake of a nurse. It had been previously used, in lotions of the strength of gr. ij or iij to the ounce of water; and with little advantage. Observing that the empirical remedies said to have succeeded, were, as I considered them, immoderately strong, I furnished the nurse with a common solution of sulphate of copper, and with a vial containing 72 grains of the sulphate in an ounce of water, for the purpose of being progressively added to the others at different periods. This stronger solution was applied, by mistake, instead of the diluted one; and it was the first remedy which had produced a rapid tendency to a cure. I finally settled down, after various trials, in the employment of the following:

"℞. Sulph. Cupri, . . . . . . . . . . ʒij.

Pulv. Cinchonæ, . . . . . . ℥ss.

Aquæ, . . . . . . . . . . . . . . . ℥iv. *m.*

"S. To be applied twice a day, very carefully, to the full extent of the ulcerations and excoriations." 20.

The cinchona is not absolutely necessary; but is useful in retaining the sulphate longer in contact with the gums. Simple ulcerations and small gangrenes yielded promptly to this remedy. The early extraction of the teeth was important—a separation of a portion of the periosteum from the fang, within the socket, was universally found, whenever the tooth was loose. When the tooth was extracted the lotion could be applied, and "the sanatory effect was surprisingly prompt."

In the New Dictionary of Medicine, volume 10, there is a description of this disease by Marjolin, to which we may refer our readers. It appears that this affection has been very fatal in the Parisian hospitals. The actual cautery has been employed with success, in several instances; the muriatic acid was the most general remedy. Marjolin avers that he cured three cases by the mere application of common salt.

---

24. PHAGEDENIC ULCERS.*

Mr. Babington, in his official situation at the Lock Hospital, has opportunities of seeing the various forms of syphilitic disease on an extensive scale. We hope he will, from time to time, communicate the results of his experience through the excellent channel which is now opened to the medical officers of public institutions. Three cases are detailed in the last number of our esteemed cotemporary, illustrative of phagedenic ulceration, and the different modes of treatment occasionally required.

The first case was treated with mercury. A young woman was admitted on the 29th January, 1825, with a sloughing sore that had destroyed the extremity of the urethra, extending up to the clitoris, and spreading rapidly. Its surface was dark, and the surrounding parts tumefied and of a bright red colour. It commenced a fortnight previously, near the orifice of the urethra, and thence extended with rapidity. She was ordered five grains of blue pill twice a day—an aqueous solution of opium and bread poultice to the ulcer—and fumigation with cinnabar. The pain was not relieved, and the fumigation distressed her. The blue pill was continued, (omitting the fumigation) and one grain of opium given every four hours. This relieved the pain, but produced no change in the sore. On the fifth day, the gums were tender, and then there was a manifest improvement in the sore. The medicine was continued, with a diminution of the opium. On the seventh day, the sore was clean, the tumefaction nearly gone. The opium was omitted altogether. The progress of the cicatrization was regular but not rapid, as the mercurial action could not be kept up in a sufficiently free manner, owing to an ulceration round one of the dentes sapientiæ. It was necessary to compensate for deficiency of effect by prolongation of the course. In a month the sore was quite healed, but the blue pill was continued for another fortnight.

*Case* 2. S. Starkey, aged 20 years, was admitted on the 21st April, 1825, with a large foul sore on the inside of the right thigh, not far from the pudenda. It was larger than a cleft orange, very dark and foul on the surface, and surrounded by an inflamed border of a dark red colour —edges tumid and everted. The pain was constant and severe, almost entirely preventing sleep. The ulcer had commenced three weeks previously, in the shape of a common pimple. Its increase had latterly

---

* G. Babington, Esq. Surgeon to the Lock-Hospital. Med. Journ. September, 1826.

been rapid, with proportionate pain. She had a discharge, and also a small sore on the labium pudendi. One grain of opium every four hours—aqueous solution of opium—bread and water poultice. 22d. Pain subdued. 23d. Free from pain—aspect of sore improving—opium only every six hours. 26th. Healthy granulations—opium ter in die. 29th. The pain returned, and the sore again unhealthy. Opium every six hours. The sloughing was soon arrested by the opium, but the sore was languid, and the granulating process slow. The opium was continued, and a pint of the Lisbon diet drink given daily. *May* 12th. A violent diarrhœa came on, supposed to be owing to the long-continued use of large doses of opium. This was accordingly omitted—and some tincture of ginger was added to the sarsaparilla. The disorder of the bowels ceased; but the pain in the sore returned with as much violence as ever; and it began again to slough, with great rapidity. The tincture of opium was now substituted for the substance, and given in doses of twelve minims every six hours. The disorder of the bowels did not recur—the pain soon ceased—the sore assumed a healthy aspect—and the general health improved under the sarsaparilla. At the end of a month, the ulcer was reduced to the size of a shilling, but it was not healed till the 21st of June.

*Remarks.* As this sore was of a syphilitic nature, as much as the one previously described, we see no good reason why a similar treatment should not have been *tried*, at least during some part of the time. It cannot be averred that mercury would here have aggravated the complaint at any period, since there was no attempt made to administer it. The case proves, no doubt, that these sores will heal under opium and sarsaparilla—or indeed without any other medicine than that which may be necessary to regulate the bowels, with low regimen. But the question still recurs, which mode of treatment is the best, not only for the immediate cure, but for the subsequent security?

*Case* 3. John Bourke, aged 24 years, admitted 14th Jan. 1826, with an extensive ulcer in the right groin, formed by a bubo that had burst a month previously. The surface was unhealthy, the edges becoming jagged and irregular. There was another ill-conditioned sore on the scrotum, and also a primary one on the glans penis. He appeared to have passed through a course of mercury before admission; "but as the effect had been violent, it was uncertain whether it had been conducted with sufficient regularity." At this time, however, the state of the sores and of the general health precluded mercury—at least for the present. A pint of sarsaparilla decoction daily—and linseed poultices to the sores. 21st. The ulcers shewed some disposition to spread—tongue furred—pulse quick—skin hot—whole system disordered. Sarsaparilla discontinued—to take saline draughts, with 30 minims of the vinum antimonii, and a drachm of sulphate of magnesia three times a day. 24th. The pulse less active, but the tongue still foul, and the sores sloughing. The mistura cinchonæ with half a drachm of the sulphate of magnesia thrice a day—treacle to the sores, and over that a bread poultice. 28th. Sores rapidly extending and sloughing—tongue furred, and assuming a darker appearance—restless and sleepless. Medicines to be continued. 31st. Sloughing process still continuing with rapidity—hæmorrhage this morning from the sore in the groin to a considerable extent, but not from any of the large blood-vessels. The pulse is greatly depressed—countenance pale—tongue dark and dry. Bark mixture, tincture of ginger, every six hours—muriatic acid, diluted, to the sore—fifteen grains of Dover's powder at night. *Feb.* 3d. Sloughing continues—muscles of the thigh exposed—tongue still black—pulse depressed. Two grains of the sulphate of quinine were now given every three hours in pills, with a draught of infusion of roses well acidulated.† *Feb.* 11. The sores have been spreading less rapidly—surface red, but granulating slowly—pulse weak—tongue clean—appetite good. The quinine was continued till the 8th of April, and the sores were all healed by the 21st May.

There can be little doubt, we think, that the quinine, in this case, was the principal mean of saving the man's life. It is to be deplored that adulteration of this valuable medicine is now carried to a great extent. We understand that preparations are making on a large scale by a house in the city for the manufacture of the sulphate of quinine. The price of spirit, however, will be a great draw-back on the British chemist.

---

25. SURGICAL REPORTS OF M. ROUX.*

The reporter, on this occasion, is M. Velpeau, "Chef de Clinique," who avers, "without fear of contradiction, even by foreigners," that the Parisian hospitals constitute the finest philanthropic institution in the world—"*la plus belle institution philanthropique qui existe dans le monde*." We are too polite, even as Englishmen, to contradict our Parisian confrere, but we cannot help thinking that the asseveration would have sounded quite as well in the mouth of a foreigner, as in that of a "chef de clinique." M. Velpeau observes that, notwithstanding the vast mass of records carefully compiled in these hospitals by diligent and zealous students, the great majority of them are entirely lost to science. The registers, it is true, are open to all enquirers, but who can wade through the chaos of histories and details in search of that which he wants? On this account M. Velpeau thinks that the only way to render hospitals useful to the profession at large is to charge some individual with the task and responsibility of reporting the practice of one medical officer—"si, dans chaque hôpital, quelqu'un se chargeait de faire connaitre les principaux faits qu'on

---

† We would recommend that the sulphate of quinine should never be given in pills, especially in such states of the constitution as above described, where the digestive power must be almost annihilated. Where can there be a more excellent form than the quinine in the acidulated rose-leave draughts?

* Hôpital de la Faculté. Archives, Juillet, 1826.

y observe, et d'exposer les regles de pratique adoptées par chaque chirurgien," &c. We are quite convinced that this is the only plan of getting a fair, full, and impartial report of hospital cases. It is not every raw pupil who runs round the wards of an hospital, that is fit to draw up the histories of diseases therein contained. It must be an intelligent, well-qualified, accredited, and responsible pupil, having, at all times, access to the patient, to his nurses, and to the dispensary of the hospital, who can investigate the previous history, and faithfully record the daily progress of a disease. A mere catalogue of certain prominent symptoms and principal prescriptions is not the history of a case, and never can be a record of much use to the public, for the truth of which we appeal to every man of clinical observation. We perfectly agree with M. Velpeau, therefore, in the sentiments which he expresses at the commencement of his report.

The hospital of which we are now speaking is destined almost exclusively for operative surgery, and it is here that pupils are taught and permitted to operate on the *living* body, under the guidance of M. Roux. We wish that something of this kind could be effected in this country. But the economy of hospitals being more a *parish* than a government concern, in this land of liberty, we fear the thing can never be done. In the reports which M. Velpeau makes, he is sanctioned by his superior in mingling his own personal observations and experience, by which the said reports may be much enriched.

1. *Excision of the Tonsils.* M. Roux prefers a straight probe-pointed bistoury to the scissors. While the jaws are kept asunder by means of a piece of cork, a hook is plunged into the enlarged tonsil, and it is cut out from above downwards, by the bistoury.

2. *Fistulous Ulcers.* M. Roux evinces some peculiarities of practice in fistulæ. When sinuses, for instance, run along under the integuments on the extremities, or elsewhere, he not merely lays them open their whole length, but excises a portion of skin over their track, by which means, he avers, they heal much better. So in fistulæ about the anus:—he gives himself very little trouble in searching for the opening into the gut—for sometimes this is several inches from the anus—he therefore pushes the bistoury through, and divides the fistula. If there are several sinuses about the verge of the anus, he slits them open in all directions, and cuts away the flaps of loose skin, making a large sore, which, however, soon heals. It is in this way he treats, with great success, it is said, those fistulous openings which so often remain in the breasts of females after milk-abscesses.

3. *Deep-sealed Abscess.* Inflammation of the cellular tissue round the femur near its tibial extremity is a dangerous disease. The anatomical disposition of the parts favours the production and collection of large quantities of matter that spread far and wide, separating the muscles and denuding the bone. Death is generally the consequence.

M. Roux has proved that some cases may be saved by very bold incisions, and subsequent tight bandaging.

*Case.* A boy, 12 years of age, was brought into the hospital on the 10th of December, presenting an enormous swelling of the right thigh, of a fortnight's duration. Poultices were applied for a few days, when M. Roux thought he could perceive some obscure and deep-seated fluctuation. An incision, four inches in length, was made parallel with the sartorius, and terminating a few inches above the knee. He divided, in succession, but cautiously, the skin, the cellular membrane, the aponeurosis, and the vastus internus muscle, when the knife entered the abscess, and nearly two pints of matter were discharged. The next day a counter-opening was made on the opposite side—some bands (brides) divided—and a large seton passed through behind the femur. The suppuration was great, but the little sufferer's strength was kept up, and, by a judicious use of bandages afterwards, the case was brought to a favourable conclusion.

---

### 26. DISEASE OF THE TESTICLE.*

The exact nature of swellings in the testicle, no more than in any other part, cannot always be ascertained by the most experienced surgeon, till the scalpel unravels the mystery.

A man was admitted into St. George's Hospital, on the 12th July, 1826, the left testicle being enlarged to the size of a goose's egg, and of an irregular oval form—tumour ponderous, and hard to the touch, with here and there some indistinct feeling of fluctuation—no pain or tenderness in the testicle itself—slight pain in the back. The complaint began three months previously, without pain, and gradually increased to its present state. A puncture was made with a sharp instrument, but no fluid was found. The patient was next put on a course of mercury, till the mouth became sore, but without benefit, as was, indeed, prognosticated by Mr. Brodie. The testicle was therefore removed in the usual way, the vessels being secured by separate ligatures, after the division of the chord. In two or three days from the operation, there were symptoms of thoracic inflammation, which gave way to the usual remedies.†

On dissection of the testicle, the morbid structure was found to consist of two parts—first, a multitude of small hydatid-like cysts, containing serum—secondly, a grey, firm substance, of a somewhat ligamentous structure, in which the cysts were imbedded. The glandular structure of the testicle was entire and unchanged, but occupying every where the circumference of the tumour, and forming a thin layer

---

* Mr. Brodie, St. George's Hospital, Med. Journ. September, 1826.

† So it is stated in the report, brought up to August 13th. But we have learnt that the man died about a fortnight after the date of the report, of intense peritoneal inflammation. It is worthy of remark, that some of the mesenteric glands were found to be affected with the same disease as that in the testicle, forming a strong presumption that there prevailed a constitutional tendency to this morbid process—a fact denied by some very high surgical authorities in this metropolis.—*Ed.*

on its surface. The epididymis was also sound, as was the spermatic cord.

In the same journal Mr. Brodie has given us some particulars of a fungous tumour on the back, arising from a scratch. The patient was a man about 54 years of age, admitted into the hospital on the 19th April, 1826. Having been once severely flogged in India, he carried the scratch of the boatswain's *cat*, ever since, in the form of a cicatrix, which, however, was unaccompanied by any pain. His health was good, until he accidentally scratched himself with his finger-nails, about four months previous to the date above-mentioned, at which time, the part bled and afterwards degenerated into a sore, whence a fungus sprouted out, attended with severe lancinating pain and offensive discharge. The tumour had recently enlarged considerably, and the man had lost flesh. The surface of this fungus was irregular, and divided into a multitude of small portions, giving the whole an appearance of a large warty excrescence. The skin, in the neighbourhood, was of a dark red colour, and covered with small tubercular projections. On the 24th April, the tumour, with a small portion of the surrounding skin, was removed by the knife. There was not much hæmorrhage, and the part was dressed with dry lint. The wound healed rapidly, and the patient was discharged, cured, on the 7th of June.

We should be glad if Mr. Abernethy, Dr. Good, or any other distinguished classifier, would give to this tumour—

"A local habitation† and a name."

Considering the number of warty excrescences which bristled on the back of the tumour, we believe the name of "*cat o' nine tails*" would be as proper as any we could give it. At least, if Dr. Good will turn this into Arabic, it will answer the purpose perfectly well.

---

27. POLYPHAGIA.*

Jacques de Falaise, æt. 62, a stone-cutter, in the quarries of Montmartre, and of a very muscular habit, had been always blessed with a tolerable appetite, not, however, an unnatural one. One day, after drinking deep, he was challenged to swallow a bullfinch alive, which he did instanter. After this feat, he was strongly urged by his comrades not to bury such talents in obscurity, and his fame reaching the ears of one of those theatrical gentlemen, yclept managers, who love to encourage merit in every shape, he was engaged at one of the Parisian playhouses for 400 fr. per annum to swallow publicly whatever was put before him. He commenced his career by *bolting*, for eat them he did not, birds, flowers, even pieces of money. Being advertised once to swallow 60 five-franc pieces, he proceeded as far as 40 to the no small consternation of the contributors, who saw enough to prevent them from

---

† We suppose, from the emphasis, which is laid on the expression "*at present*," that the cast of this tumour is not meant to remain where it now stands—in the museum of the College of Surgeons.

* M. Beaudé. Hôpital Beaujon. Nouv. Biblioth. Juin, 1826.

risking the remainder. Frogs, cray-fish, eels, adders, all came alike to this living Charybdis, but the pain arising from the motions of the animals in his stomach induced him latterly to crush their heads with his teeth before gorging them. This could not continue for ever; he entered the hospital Beaujon for a gastro-entérite, under which he suffered for several months. On his discharge, however, he resumed his old trade; but after a time, what with the effects of his "professional duties," and what with chagrin at being robbed of his savings, he re-entered the hospital with a more dangerous gastro-enteritis than before.

After a long and painful illness he recovered, and accepted a situation in the hospital. His temper now became soured, and, on the 30th March, after spending the evening in a tavern, he hung himself.

*Dissection, 24 hours after death.* The pharynx and œsophagus were much enlarged. At the cardiac extremity of the latter were three small tubercles, arising from, and apparently of the same structure as the mucous membrane.

The stomach was much distended by a nauseous mixture of half-digested aliment, wine, and three playing cards, which he had probably swallowed at the tavern. The mucous coat of this organ was sound, but the muscular fasciculi were developed in an extraordinary manner, especially towards the cardia. The pyloric orifice was enlarged.

The ileon, at its lower extremity, presented three or four patches, where the mucous membrane appeared to be destroyed, and the other coats were so thin as to be quite transparent. In the cæcum were numerous large cicatrices of ulcers, originating, no doubt, in the gastro-enteritis, which the patient had laboured under. The liver was very large.

Such are the principal circumstances of this case; a remarkable one as instancing the manner in which the digestive organs may be set at defiance, one might almost say, with impunity. Is it not strange that after throwing into the stomach, as this man did, literally whatever came in his way, there should be found, on dissection, merely a few remains of old ulcers, the frequent consequences of a common fever? So it is, however, that, while one man can scarcely take a scruple more than his allowance, without suffering severely, another can swallow five-shilling pieces with little apparent mischief.

---

### 28. URINARY CALCULUS.

The instances on record of extraction of urinary calculi from the female bladder, by dilatation of the urethra, are now numerous. Mr. Chevalier has added one to the list. The patient was a girl, only 16 years of age, who applied at the Westminster Dispensary, in January, 1825. She had been complaining for several years, of lancinating pains about the bladder, especially when making water, (which was sometimes bloody,) together with pains in the loins and other symptoms which rendered her life miserable. She had been sounded at the Middlesex Hospital, and a calculus detected, but she would not submit to an operation. Mr. C. felt the stone, both with the sound and with the finger introduced "per vaginam." An attempt to introduce the smallest sized dilator urethræ failed, and a phimosis dilator was next employed, and opened to the extent of three-fourths of an inch. We need not detail the many processes which were gone through, no doubt with all possible dexterity, till the enemy was dislodged—and a very rugged enemy it was, the nucleus being composed of lithate of ammonia and oxalate of lime, surrounded by a mulberry calculus, projecting in noduli of more beauty than kindly qualities. The calculus is more than an inch and a quarter in diameter, and the safe extraction of such a monster does great credit to Mr. Chevalier. The inflammatory consequences of the operation were not more severe than might have been expected, and were so judiciously combated that, in a short time, the urethra had nearly regained its natural dimensions, and the healthy function of the bladder was apparently restored. But a sinister train of symptoms afterwards occurred. Incontinence of urine first appeared, and next a complete retention, till a slough came away, and then incontinence of urine ensued. It took upwards of three months for her complete recovery from the incontinence, and a still longer period elapsed before she was able to retain more than half a pint of water at one time in the bladder. At length she recovered completely.

The history of this, and, indeed, of some other cases of extraction of calculi, through dilatation of the female, impresses us, as it has done Mr. Chevalier, that (where the calculus is large) no patient would submit to it, if previously aware of the amount of suffering which was to be endured. Nothing but the great risk of subsequent incontinence of urine can make it desirable to prefer the dilator to the knife. Mr. Chevalier respectfully suggests to the profession the high operation in preference to either the common operation of lithotomy or dilatation. He observes that, "with proper management, it must be a safer operation in the female than in the male," and he knows of "nothing to prevent its being perfectly safe and perfectly successful"—while it is only of a few minutes duration. We hardly expect that this suggestion will be acted on; nor do we think that the high operation, either in male or female, can ever be rendered so safe a measure as Mr. Chevalier seems to anticipate. The difficulty and pain of extracting large calculi, per urethram, must make it a great object with practitioners to perform this operation at an early stage of the growth of these foreign bodies; and, to this, they should look in time.—*Med. Journ. September*, 1826.

---

### 29. M. LISFRANC ON SCIRRHUS.*

M. Lisfranc has given some clinical reports on scirrhus of the female breast, in the treatment of which he applies the same principles as are developed in his report on white swellings, already published in this Journal. The leechings are managed in the same manner, and the same

---

* M. Lisfranc. La Pitié.—Archives, Juillet.

views are entertained respecting the excitant and sedative effects of their application, according as a small or a large number are used. We shall here state a case which is not devoid of interest, on several accounts.

*Case.* Catherine Martin, 19 years of age, of lymphatic temperament, had not been regular for 15 months previously. In April, 1825, she felt some pain, and perceived a small hard tumour in the right breast. This increased in size, and a surrounding engorgement took place, till the breast attained twice the size of the other. In this condition the breast felt as consisting of several tumours—or, in other words, *tuberculated.* There were lancinating pains—two or three small abscesses formed, and on the 9th September she entered La Pitié, the breast being then hard, shining, very large, tuberculated, adherent to the chest, the nipple sunk and non-apparent, skin discoloured and adherent to the subjacent parts, discharge of ill-conditioned pus from three openings, intermittent pains of a severe and lancinating character, with sense of burning heat—in short, the general symptoms presented where amputation of the breast is recommended. M. Lisfranc not wishing to have recourse to the knife, without a trial of other means, directed fifteen leeches to be applied, and then emollient cataplasms. During each of the ensuing four days the same number of leeches were applied, and then six were applied every fourth or fifth day. In this way ten applications were made altogether, and the poultices were continued. No benefit resulted from this plan, and five new abscesses opened, discharging bad matter, mixed with a cheesy substance. As soon as one abscess healed, another broke out in its neighbourhood. Mercurial frictions were now tried, and were followed by increase of inflammation and swelling. The frictions were discontinued, and poultices repeated. On the first of October, frictions with the hydriodate of potash, in the quantity of half a drachm at a time, were commenced, and in a very few days, it was observed that the volume of the breast was diminished. The quantity of iodine was then increased to a drachm—and, in ten days, to a drachm and a half. Under this treatment the breast diminished in a rapid manner, and became soft in proportion—the pains ceased—the discharge diminished—and, by the end of October, the two breasts were of equal size, and the patient left the hospital cured.

*Remarks.* This case shews that when antiphlogistic measures have failed, we are not to have recourse to the knife immediately, as the disease may still be scrofulous and not cancerous, as was evidently the case in the present instance, notwithstanding the lancinating pains, the disappearance of the nipple, and other suspicious symptoms. The treatment pursued was, we think, judicious. The iodine frictions, we have no doubt, were much more efficacious after such repeated leechings, than they would have been, had no such preliminary treatment been employed.

---

### 30. INJURIES OF THE HEAD.

In the last Number (3, new series) of the Medical and Physical Journal, Mr. Jeffreys has detailed four cases of injuries of the head treated at St. George's Hospital. We shall give a short sketch of three of these.

*Case* 1. A coachman fell from his box in a state of intoxication, and was brought into the hospital stupid and insensible, with a small wound, but no fracture, of the left temple. The wound was dressed, and he was ordered a senna draught next morning. He slept sound all night, and was still heavy and drowsy next day. The pulse being now full and quick, he was bled to 12 ounces, and had a dose of calomel and James's powder, followed by a black draught. The pulse sunk after bleeding, but rose in the evening, and he was bled again, and well-purged. It was found that he had lost the sense of hearing in both ears. Blisters, low diet, and frequent purging had little effect, and he left the hospital, without recovering the auditory function.

*Case* 2. A man, sixty-three years of age, fell down stairs, and was picked up insensible, presenting a lacerated wound of the scalp, three inches long, over the right parietal bone, which was not denuded. When brought to the hospital he had nearly recovered his senses. A senna mixture every four hours till it operated. Passed a good night; but next day (April 6th) he complained of pain in his head—had dry, furred tongue—quick, full pulse. Twelve ounces of blood from the arm—bowels to be kept open—low diet. By these means the head-ache was relieved, and every thing seemed to be going on well till the seventh day, when he fell into a stupid, semi-comatose state—and had a dry brown tongue, with quick, but not full pulse. Bled to ten ounces—purgatives. Next day (8th from the accident) he continued in the same state—pulse quick and intermitting—tongue very dry—involuntary discharge of urine. Venæs. ad ℥x. soon after which he became delirious, and so violent that it required three men to hold him. A large blister over the head. In the evening more tranquil—answers questions rationally. Next day, the improvement continued. On the 10th day, there was return of pain in the head—pupils sluggish—pulse quicker than natural. Bled to ten ounces. Was more sensible in the evening, but had a restless night. *11th day.* He was still sensible, and the wounds looking well—pulse very low and irregular—in the night delirious. *12th day*, comatose. *13th*, erysipelas of the face, and in the evening he died.

*Dissection.* On the side *opposite* to the wound there was found an extensive fissure in the parietal bone, descending through the temporal bone, and terminating at the cella turcica. It also passed across the sagittal suture to the middle of the right parietal bone. Upon the dura mater, on the left side, under the fracture, was a deposit of coagulated blood, of the thickness of a dollar, and two inches in diameter. The dura mater itself was not inflamed; but the tunica arachnoides was injected with *red blood;* and between it and the pia mater there was a copious effusion of coagulable lymph, and some serum. The sternum was fractured.

*Remarks.* It is not likely that any treatment would have saved this case; but we submit it to Mr. Jeffreys' better judgement whether, in such cases, a more copious depletion from the vascular system, *in the beginning*, might not save a good deal of mischief subsequently. In concussions as well as fractures of the cranium, the moment re-action begins to shew itself, we can hardly be too active in our depletive measures; for surely it is much more easy to prevent inflammation, or to arrest its progress in its first stage, than control it afterwards, when the sensorial powers are oppressed by the extent of the disease. There could not be more unequivocal evidence of intense meningeal inflammation than the foregoing case presented; and we doubt whether the abstraction of twelve ounces of blood in the first seven days was sufficient to prevent the terrible phenomena which then developed themselves. Nothing can be more insidious than inflammation within the cranium after concussions and fractures—and the danger is far less in erring on the side of copious early depletion, than in the opposite error. We cannot but think, also, that surgeons are remiss in not using auxiliaries to depletion with a more liberal hand. Antimony and colchicum control vascular excitement in no mean degree, and should never be overlooked in such cases. Blood-letting is a *sine qua non*, but it is not the *unicum remedium*.

Passing over the third case, we shall advert to the fourth.

*Case* 4. A youth, 16 years of age, received a kick from a horse in the face, by which he was stunned for about five minutes, and then walked to the hospital. On his arrival he was sensible and collected, but blanched from the hæmorrhage. There was a semi-circular wound across the right eye-brow and root of the nose. The ossa nasi were broken to pieces, and driven inwards, and there was a fracture of the superciliary ridge extending into the frontal sinus and within the orbit, with very slight depression of the orbital portion of the superciliary ridge. On sponging the wound, a very small portion of medullary matter was perceived. The wound was dressed—a spirit lotion applied—and a senna mixture prescribed. The accident happened at 7 o'clock in the morning. At 2 P. M. he complained of pain across the forehead, and was beginning to be restless—pulse 80. Bled to 12 ounces. At 7 in the evening, he had a cathartic of calomel and jalap which operated, and he slept much in the night, occasionally awaking in a state of agitation. Next morning he was collected and quiet, complaining of slight pain in the forehead—tongue rather dry—pulse 108. He was again bled, and purgative medicine prescribed. Towards evening he got restless, and talked incoherently. At half-past six he became unmanageable, with oppressed breathing, and suddenly expired.

*Dissection.* The fracture extended through the frontal sinus, and along the superciliary ridge into the orbit. The orbitar process of the os frontis was broken and splintered, some of the fragments being driven through the dura mater, and lodged in the substance of the anterior lobe of the brain.

We will make one more remark before we take leave of this subject—namely, that we consider it useless, perhaps injurious to bleed *before*

any reaction takes place, in cases of concussion or fracture, because we believe that it thus rather tends to increase than to diminish subsequent excitement. But whenever reaction commences, then we should deplete freely, and meet every rising of excitement by the most vigorous means. Local depletion by leeches, and artificial cold, to the head are not sufficiently used by surgeons in this country.

### 31. CURIOUS CASES OF PARALYSIS.*

If there is any one part of the human fabric more involved in obscurity than another, it is the nervous system. Much as has been done in this country by Monro, Philip, and Bell, as well as by Majendie, Spurzheim, and others, on the Continent, it must be confessed that more, much more, remains behind, before we can get any comprehensive views of that stumbling-block to the physiologist, aye, and to the practical physician—the nervous system. It is only from an extensive collection of well-authenticated facts that any tolerably accurate inferences can be drawn. To this end, M. Velpeau, a man of talent and eminence in the French capital, has published a memoir on some extraordinary cases of paralysis, the gist of which we shall lay before our readers.

*Case* 1. Huette, æt. 52, a nurse, of strong constitution, at the age of 30, suffered from malignant fever, after which she continued well till her 48th year, when her menstruation became irregular, and phthisical symptoms succeeded. In the morning of the 12th January, 1825, without any previous disturbance whatever of the intellectual functions, she was suddenly seized with hemiplegia of the left side. On her admission into the Hôpital de la Faculté, on the 13th, the limbs of the left side appeared completely paralyzed; the tongue and head were turned rather to the left; urine abundant, and both it and the motions were passed without the knowledge of the patient; abdomen somewhat swollen and tender; face livid; little thirst; pulse regular; respiration laborious; the intellectual functions preserved their integrity. From this day she became weaker and weaker, and in the evening of the 18th, she died, the right side still maintaining its sensibility.

*Dissection, 36 hours after death.*—The left arm and both lower extremities were œdematous. On opening the cranium, the longitudinal sinus was found congested—the pia mater was injected. The mass of the brain was found remarkably firm—the commissura mollis almost as dense as the skin, and very elastic.—The ventricles were dry, and the plexus choroides had contracted a few insignificant adhesions with the surrounding parts.

About the middle of the cervical portion of the spinal marrow there appeared a cleft about an inch in length; from the left side of this a smaller transverse fissure proceeded to the extent of about three lines. On turning up the external border of this fissure, there was seen a cavity, three inches in length and two or three lines in breadth, in the centre

* M. Velpeau. Clinical Report of the Hospice de Perfectionnement. Revue Médicale, May, 1826.

of the right column of the spinal marrow; this cavity contained a reddish, diffluent, and almost purulent kind of bouillie, apparently the grey substance of the marrow converted into pus; its walls were firm, and about a line and a half in thickness. In the left column, opposite the first, was found a second cavity, an inch long and a line broad, which contained a semifluid matter, which appeared to be the cineritious substance slightly softened. The spinal marrow, especially the cortical portion, was rather hard. It may be mentioned that the dura mater in the lumbar portion of the vertebral column presented three yellowish, and seemingly osseous patches in its substance.

*Remarks.* The hemiplegia in this case, observes our author, is not explained satisfactorily, either by the pathological condition of the head or the spine. A woman is seized suddenly with total paralysis of the left side, and, in six days from the seizure, she expires. The intellectual functions have never been affected, and the brain is found, on dissection, to be much firmer than usual. Another person dies insane, the surgeon comes and finds, perhaps, the brain preternaturally firm, and this he sets down as the undoubted cause of the insanity: so that what has no effect at all on the faculties of one, is affirmed to destroy the senses of another. Look to the spine—the paralysis was of the left side only, and yet disorganization was found both in the right and the left column of the medulla. Some physiologists are of opinion that there is decussation of these columns, and M. Portal relates a case in support of it.

A woman had long been afflicted with hemiplegia of the left side, and died comatose. On examination, he found the arachnoid and pia mater much inflamed in the loins; the spinal marrow reddened and soft on the right side, apparently quite healthy on the left.

Now, assuming that there is decussation, the disorganization in the *right* column will explain the hemiplegia of the *left* side, but the *right* side ought also to be affected, for the *left* column of the medulla was disorganized likewise, though certainly not to the same extent. Again, it is difficult to account for the suddenness of the attack, and the want of previous symptoms, for both the morbid processes in the cranium and vertebral column must have been slow ones.

*Case* 2, *from Portal.* The Marquis de Causan became affected with tingling in the fingers of the right hand, and toes of the right foot. This gradually increased to paralysis, first of the right side, then of the left; sight and hearing were the next faculties that were destroyed—deglutition became difficult; the respiration became oppressed, the pulse sunk to 40, 30, 10 pulsations in the minute; at last life's taper was slowly burnt out. On dissection, the medulla of the cervical portion of the column was found hardened, and even cartilaginous. Every other part was natural.

*Remarks.* Here we see that the very cartilaginous condition, in different organs indeed, which had no effect upon one patient, was the cause of all the symptoms in the other. Even here there are some anomalies. How is it that the nerves of sight and hearing, which do not depend so directly upon the spinal marrow, were destroyed long before the respiratory nerves, which apparently have an immediate dependence on the medulla ? Mr. Charles Bell's view of these nerves would seem, in some degree, to explain the matter, for he makes them arise from a column quite distinct and separate from the common voluntary nerves.

*Case 3.* This is related by M. Bretonneau, a physician whose name is a sufficient guarantee for its authenticity.

Mad. M. was seized with paralysis of the little finger of the left hand, which gradually extended to the whole of that side ; the right side became similarly affected, with the exception of the thumb and two fingers. The whole body was thus stricken—the tongue was motionless—deglutition extremely difficult, her intellectual functions remained unimpaired, and she could express her wants by the thumb and fingers of the right hand, the motions of which continued to the last. So she died.

*Dissection, 30 hours after Death.* The only discoverable lesions in the brain were between two and three ounces of serum in the ventricles, and, on cutting the tuber annulare, there was seen a rust-coloured spot, about three lines in breadth and one in depth ; its circumference was irregular, and it lost itself insensibly in the nervous matter. No other disorganization could be found on the minutest examination of the brain, spinal marrow, and nerves. One third of the left lung was hepatized, and under that part of the peritoneum which covers the uterus, two small, white, fibrous tumours appeared.

*Remarks.* Here, again, we see the mysteries of the nervous system. In one case there is disorganization of one column of the spinal marrow, and yet no paralysis follows ; in another, there is total paralysis, and the spinal marrow remains perfectly sound. A question arises, was the patch observed in the tuber annulare the cause of all the symptoms ? M. Velpeau thinks not, and, indeed, it would be difficult to support such an opinion. The small quantity of fluid in the ventricles cannot be considered of much importance. The continuance of motion and sensibility in the thumb and fingers, whilst the arm was totally paralytic, is curious, and not easily explicable. M. Ollivier relates a case of a woman who was quite paralytic, with the exception of a zone, of some inches extent, in the thoracic region. The same author mentions an instance, at the Salpêtrière, where a woman lost the sensibility of the whole skin, except just a small patch on the right haunch. M. Rostan, at the above-mentioned hospital, pointed out a case to his pupils, in which only a small space, an inch or two in diameter, preserved its sensibility, whilst the remainder of the limb was quite paralyzed.

From these, and, indeed, numerous other facts, it is clear that the extremity of a nerve may preserve its functions, although the parts intervening between that and the origin may be totally paralyzed. It appears, too, looking at these cases, that the loss of function in the nerves may be complete, whilst there is little or no derangement of structure; and, on the other hand, as in the first case, that there may be structural disease without functional disturbance.

---

### 32. DISEASE OF THE HEART.*

We shall have occasion to make a remark or two on this case, after we have laid a brief account of it before our readers.

*Case.* James Fisher, aged 36 years, was admitted into the Middlesex Hospital on the 5th April, 1826, presenting slight rheumatic symptoms. Aperient medicine was given him, and some colchicum in camphor mixture every six hours. The rheumatic symptoms were soon relieved, but the skin became yellow, and he complained suddenly of pain at the scrobiculus cordis and right hypochondrium, augmented on pressure. These symptoms rapidly increased—the respiration became difficult—and, in short, "the patient evidently laboured under an attack of acute hepatitis." He was bled, and soon after fell asleep; but died suddenly at ten o'clock the next morning.

*Dissection.* Skin yellow—liver much enlarged and of a purple colour, exhibiting, when cut into, a firm, granulated texture, from interstitial deposition. "The heart was of unusual size, *and weighed two and a half pounds.*" The coronary arteries were thicker in their coats than natural, but not ossified. "The right auricle and ventricle were passively dilated, and *the tricuspid valve so diseased as to be incapable of duly performing its office*—in short, the right auricle and ventricle were as one large and flabby pouch, stuffed with a dark coagulum, and their structure corresponded to the softening of the heart described by Portal." The left side of the heart presented a striking contrast—its muscular walls being in a state of hypertrophy. "The aortic valves were slightly diseased." The lungs were adherent to the parietes of the thorax, and presented considerable effusion of serum into their substance. "It is *remarkable*" says the reporter, "that such extensive disease of the heart should have existed without the occurrence of *any symptom during life*, indicating its presence."

It would, indeed, have been *remarkable* had such been strictly the fact. But no mention is made of any examination, *during life*—nay, there is intrinsic evidence in the case that no examination was made. It is hardly possible that any man having the slightest knowledge of the phenomena presented by a heart in the above condition, and more especially a man of talent like Dr. Macmichael, could have investigated the state of the chest. It may be true that the heart did not call out for the house-surgeon's assistance; but had *ear* been given to its own language, it would have spoken in language that could not be misunder-

---

* Case of enlargement of the heart *(the presence of which was not manifested by the symptoms)* and of acute hepatitis. Dr. Macmichael—Med. and Phys. Journal, August, 1826.

stood. Valvular disease, to any extent, of the heart, is characterized by such distinctive phenomena, that the merest tyro in the profession may ascertain it, if he will give himself the trouble to examine—and, without this trouble, the most talented physician or surgeon will overlook one of the most important pathological conditions of the central organ of the circulation. We shall offer an example that cannot fail to bear forcibly on this point.

At the moment we are now writing, (15th Sept. 1826) there resides in King-street, Westminster, Mr. P. a half-pay Quarter-Master in the army, who served his king and country in many a bloody battle, including, we believe, that of Waterloo. Mr. P. has been very ill for eight or nine months past, and has consulted at least a dozen of physicians and surgeons—no two of whom were agreed as to the nature of his complaint. It was hepatitis—hydrothorax—asthma—stomach disorder—hypochondriasis, and many other diseases. He was accordingly treated by each, in succession, according to the view entertained of the malady, and yet without any material alleviation, but, on the contrary, increase of his sufferings.—A single examination of the chest, by percussion and auscultation, disclosed the existence of organic disease of the heart, with its usual consequences, effusion, &c. &c. To ascertain this, it required very little discrimination in the investigator. Any practitioner could have done the same without difficulty, provided he merely took the trouble to place his ear in contact with the parietes of the chest, in the proper place, and strike it in various directions with his fingers. Here, then, we have physicians of eminence—and *pure* surgeons of superlative anatomical skill, (which, be it remembered, gives them a kind of intuitive knowledge of all internal diseases) working in the dark for months and months—turning up their noses in scorn at the ridiculed process of auscultation and percussion—and treating their patient with useless, perhaps injurious, remedies. It is very true, we cannot cure this disease of the heart—but we know the *juvantia et lædentia*—we can do *some* good, and we can avoid *all* harm. Is this no advantage?—We have shewn the case to several medical gentlemen—among others, to Dr. Marshall Hall, Dr. Hewett, and Dr. Macann, who readily recognized the nature of the affection. To Mr. Lavies, an intelligent and zealous surgeon, of Westminster, we are indebted for the opportunity of investigating the complaint in the present instance. We need hardly say that the distinctive or diagnostic phenomena in this case were such as are laid down by all writers on auscultation, and which were cognizable without any difficulty. The respiratory function is much inconvenienced, there is effusion in the chest, and the train of anomalous symptoms induced by this dreadful disease in the main organ of the circulation, was well calculated to puzzle the medical attendant, until the real seat of the malady was ascertained by examination of the chest. We hope that this case will prove a stimulus to the study of auscultation, and help to dissipate the prejudices which INDOLENCE (we can give it no *gentler* term) is every day presenting against that study.

